# Supplementary material for: Systematic Analysis of Diverse Polynucleotide Kinase Clp1 Family Proteins in Eukaryotes: Three Unique Clp1 Proteins of Trypanosoma brucei
Source: J Mol Evol. 2023 Aug 22;91(5):669–86. doi: 10.1007/s00239-023-10128-x (PMC10598085; doi:10.1007/s00239-023-10128-x)
Supplement: Supplementary file 1 — (PDF 3707 KB) [file 239_2023_10128_MOESM1_ESM.pdf]

Supplementary Information

Systematic Analysis of Diverse Polynucleotide Kinase Clp1 Family Proteins in Eukaryotes: Three Unique Clp1 Proteins of *Trypanosoma brucei*

Motofumi Saito[1,2], Rerina Inose[1], Asako Sato[1], Masaru Tomita[1,2,3], Haruo Suzuki[1,3], and Akio Kanai[1,2,3*]

[1]Institute for Advanced Biosciences, Keio University, Tsuruoka 997-0017, Japan

[2]Systems Biology Program, Graduate School of Media and Governance, Keio University, Fujisawa 252-0882, Japan

[3]Faculty of Environment and Information Studies, Keio University, Fujisawa 252-0882, Japan

*Author for Correspondence:

Akio Kanai, PhD

Institute for Advanced Biosciences, Keio University

Tsuruoka, Yamagata 997-0017, Japan

Tel: +81-235-29-0524

Fax: +81-235-29-0525

E-mail: akio@sfc.keio.ac.jp

Supplementary Table S1 Summary of Clp1 query sequences used in this study

Species	Protein name	UniProt AC	Total aa length	Polynucleotide kinase region
Homo sapiens	Polyribonucleotide 5'-hydroxyl-kinase Clp1	Q92989	425	121-307
Homo sapiens	Polynucleotide 5'-hydroxyl-kinase Nol9	Q5SY16	702	306-470
Saccharomyces cerevisiae	mRNA cleavage and polyadenylation factor Clp1	Q08685	445	130-335
Saccharomyces cerevisiae	Polynucleotide 5'-hydroxyl-kinase Grc3	Q07845	632	246-450

Supplementary Table S2 Distribution of Clp1 family proteins in complete eukaryotic genomes

(a) Summary of the number of all Clp1 family proteins detected in this study (1,264 proteins).

	Taxon	Number of CDSs in RefSeq database	Number of Clp1 family protein	Number of Clp1 family protein (except splicing isoform)	Number of species registered	Number of species with Clp1 family protein	Ratio (%)
Eukarya	Alveolata	127,693	31	31	25	21	84.0
	Amoebozoa	13,315	2	2	1	1	100.0
	Cryptophyta	1,695	0	0	3	0	0.0
	Euglenozoa	67,115	33	33	8	8	100.0
	Fungi	477,076	114	113	60	60	100.0
	Metazoa	8,320,001	749	406	185	185	100.0
	Rhizaria	344	0	0	1	0	0.0
	Rhodophyta	4,803	1	1	1	1	100.0
	Stramenopiles	22,081	3	3	2	2	100.0
	Viridiplantae	3,299,379	331	209	72	72	100.0
	Total	12,333,502	1,264	798	358	350	97.8

(b) Summary of representative Clp1 family proteins used in the phylogenetic analysis (*).

	Taxon	Number of Clp1 group protein	Number of species with Clp1 group protein	Number of Nol9/Grc3 group protein	Number of species with Nol9/Grc3 group protein	Number of Clp1 family protein	Number of species with Clp1 family protein
Eukarya	Alveolata	15	15	10	10	25	16
	Amoebozoa	1	1	1	1	2	1
	Cryptophyta	0	0	0	0	0	0
	Euglenozoa	12	6	4	4	16	6
	Fungi	39	39	41	41	80	46
	Metazoa	23	20	55	53	78	57
	Rhizaria	0	0	0	0	0	0
	Rhodophyta	1	1	0	0	1	1
	Stramenopiles	1	1	2	2	3	2
	Viridiplantae	15	11	31	22	46	25
Archaea	Euryarchaeota	2	2	0	0	2	2
Bacteria	Deinococcus-Thermus	1	1	0	0	1	1
	Total	110	97	144	133	254	157

(*) To create a sequence set of 254 representative Clp1 proteins, similar sequences were removed from the 1,264 detected Clp1 family proteins with CD-HIT. We also excluded the protein sequences of splicing variants. We then added the sequences of representative organisms (Table 1) and the sequences of prokaryotes for comparative analysis.

Supplementary Table S3 List of Clp1 family proteins (both Clp1 and Nol9/Grc3 groups) used for amino acid sequence alignments

Taxon	Taxid	Species	Protein name	RefSeq ID	aa length
Euglenozoa	185431	*Trypanosoma brucei* TREU927	*Tb*-Clp1-t1	XP_843821.1	441
			Tb-Clp1-t2	XP_845487.1	423
			Tb-Clp1-t3	XP_844561.1	512
	679716	*Trypanosoma brucei gambiense* DAL972	*Tbr*-Clp1-t1	XP_011772180.1	441
			Tbr-Clp1-t2	XP_011774153.1	423
			Tbr-Clp1-t3	XP_011773013.1	512
	420245	*Leishmania braziliensis* MHOM/BR/75/M2904	*Lb*-Clp1-t1	XP_001565679.1	445
			Lb-Clp1-t2	XP_001566862.1	425
			Lb-Clp1-t3	XP_001567033.1	522
	5679	*Leishmania panamensis*	*Lp*-Clp1-t1	XP_010699796.1	463
			Lp-Clp1-t2	XP_010701260.1	425
			Lp-Clp1-t3	XP_010701413.1	522
	929439	*Leishmania mexicana* MHOM/GT/2001/U1103	*Lm*-Clp1-t1	XP_003876277.1	445
			Lm-Clp1-t2	XP_003877385.1	425
			Lm-Clp1-t3-1	XP_003877542.1	522
			Lm-Clp1-t3-2	XP_003877540.1	522
	347515	*Leishmania major* strain Friedlin	*Lma*-Clp1-t1	XP_001683978.1	445
			Lma-Clp1-t2	XP_001684846.1	425
			Lma-Clp1-t3	XP_001685005.1	522
	435258	*Leishmania infantum* JPCM5	*Li*-Clp1-t1	XP_001466257.1	445
			Li-Clp1-t2	XP_001467088.1	425
			Li-Clp1-t3	XP_001467282.1	521
	5661	*Leishmania donovani*	*Ld*-Clp1-t1	XP_003861557.1	445
			Ld-Clp1-t2	XP_003862954.1	425
			Ld-Clp1-t3	XP_003863112.1	521
Alveolata	31271	*Plasmodium chabaudi chabaud*	*Pc*-Clp1	XP_744232.1	590
	5858	*Plasmodium malariae*	*Pm*-Clp1	XP_028860391.1	633
	1120755	*Plasmodium cynomolgi* strain B	*Pcy*-Clp1	XP_004221125.1	591
	5855	*Plasmodium vivax*	*Pv*-Clp1	XP_001614946.1	562
	1133968	*Babesia microti* strain RI	*Bm*-Clp1	XP_012650399.1	494
			Bm-Nol9	XP_012648352.1	534
	5874	*Theileria annulate*	*Ta*-Clp1	XP_953879.1	493
	484906	*Babesia bovis* T2Bo	*Bb*-Clp1	XP_001610803.1	538
			Bb-Nol9	XP_001609650.1	607
	353152	*Cryptosporidium parvum* Iowa II	*Cp*-Clp1	XP_626224.1	601
	5866	*Babesia bigemina*	*Bbi*-Nol9	XP_012767159.1	737
	1537102	*Theileria equi* strain WA	*Te*-Nol9	XP_004831100.1	597
Metazoa	7739	*Branchiostoma floridae*	*Bf*-Nol9	XP_035677701.1	694
	8364	*Xenopus tropicalis*	*Xt*-Nol9	XP_002933893.2	639
	31033	*Takifugu rubripes*	*Tr*-Nol9	XP_011601118.2	701
	7918	*Lepisosteus oculatus*	*Lo*-Nol9	XP_015192803.1	562
	93934	*Coturnix japonica*	*Cj*-Nol9	XP_015738036.1	651
	7957	*Carassius auratus*	*Ca*-Nol9	XP_026092660.1	707
	219594	*Aythya fuligula*	*Af*-Nol9	XP_032057582.1	634
	9606	*Homo sapiens*	*Hs*-Nol9	NP_078930.4	702
	7757	*Petromyzon marinus*	*Pm*-Nol9	XP_032827451.1	782
	7245	*Drosophila yakuba*	*Dy*-Nol9	XP_002092281.1	1,032
	7227	*Drosophila melanogaster*	*Dm*-Nol9	NP_611084.2	995
	7240	*Drosophila simulans*	*Ds*-Nol9	XP_016027901.1	1,054
	7241	*Drosophila subobscura*	*Dsu*-Nol9	XP_034652937.1	873
	7159	*Aedes aegypti*	*Aa*-Nol9	XP_021699171.1	1,034
	2817044	*Belonocnema kinseyi*	*Bk*-Nol9	XP_033215408.1	754
Amoebozoa	352472	*Dictyostelium discoideum* AX4	*Dd*-Nol9	XP_642025.1	683
Viridiplantae	51953	*Elaeis guineensis*	*Eg*-Nol9	XP_029119051.1	407
	4615	*Ananas comosus*	*Ac*-Nol9	XP_020108251.1	394
	3847	*Glycine max*	*Gm*-Nol9	XP_003525672.1	372
	22663	*Punica granatum*	*Pg*-Nol9	XP_031406670.1	375
Fungi	4956	*Zygosaccharomyces rouxii*	*Zr*-Grc3	XP_002498342.1	624
	322104	*Scheffersomyces stipitis* CBS 6054	*Ss*-Grc3	XP_001386260.2	663
	931890	*Eremothecium cymbalariae* DBVPG#7215	*Ec*-Grc3	XP_003647416.1	633
	4950	*Torulaspora delbrueckii*	*Td*-Grc3	XP_003682747.1	623
	1071383	*Kazachstania naganishii* CBS 8797	*Kn*-Grc3	XP_022464617.1	655
	28985	*Kluyveromyces lactis*	*Kl*-Grc3	XP_453983.1	631
	559292	*Saccharomyces cerevisiae* S288C	*Sc*-Grc3	NP_013065.1	632
	56646	*Fusarium venenatum*	*Fv*-Grc3	XP_025587698.1	720
	332648	*Botrytis cinerea* B05.10	*Bc*-Grc3	XP_024547363.1	776
	510516	*Aspergillus oryzae* RIB40	*Ao*-Grc3	XP_023093528.1	816
	759273	*Colletotrichum higginsianum* IMI 349063	*Ch*-Grc3	XP_018164591.1	755
	148305	*Pyricularia grisea*	*Pg*-Grc3	XP_030986943.1	727
	367110	*Neurospora crassa* OR74A	*Nc*-Grc3	XP_959496.1	804

Supplementary Table S4 Summary of detected domains in Clp1 family proteins

Domain symbol	Protein domain name	Protein domain details	Pfam ID
	Clp1_P	Clp1 polynucleotide kinase domain in eukaryotes	PF16575
	Clp1_eN1	Clp1 N-terminal domain in eukaryotes	PF16573
	Clp1_eN2	Clp1 N-terminal domain in eukaryotes	PF16573
	Clp1_eC	Clp1 C-terminal domain in eukaryotes	PF06807
	CTLH	CTLH/CRA C-terminal to LisH motif domain	PF10607
	Clp1_aC	Clp1 C-terminal domain in archaea	*1
	Clp1_alC	Clp1 C-terminal domain in alveolata	*2
	Clp1_euC1	Clp1 C-terminal domain in euglenozoan type 1	*2
	Clp1_euC2	Clp1 C-terminal domain in euglenozoan type 2	*2
	Clp1_euC3	Clp1 C-terminal domain in euglenozoan type 3	*2
	Nol9_eN1	Nol9 N-terminal domain in metazoan (1)	*1
	Nol9_eN2	Nol9 N-terminal domain in metazoan (2)	*2
	Nol9_eN3	Nol9 N-terminal domain in alveolata	*2
	Nol9_eC1	Nol9 C-terminal domain in metazoan, viridiplantae and amoebozoa	*1
	Nol9_eC2	Nol9 C-terminal domain in alveolata	*2
	Grc3_eN1	Grc3 N-terminal domain in fungi (1)	*2
	Grc3_eN2	Grc3 N-terminal domain in fungi (2)	*2
	Grc3_eC1	Grc3 C-terminal domain in fungi (1)	*2
	Grc3_eC2	Grc3 C-terminal domain in fungi (2)	*2

Protein domains were identified using two methods: (i) searches against the Pfam database; and (ii) manual amino acid sequence alignments (all symbols are rectangles). *1, Saito et al. 2019, *2, this study.

Supplementary Table S5 Summary of Clp1 and RNA ligase family enzymes (Trl1/Rnl and RtcB) in the species examined in this study

Domain	Taxon	Species	Number of tRNAs with introns	tRNA splicing pathway		
				5'-phosphate ligation pathway		3'-phosphate ligation pathway
				Clp1	Trl1/Rnl	RtcB
Eukarya	Metazoa	*Homo sapiens*	28	◯	-	◯
	Fungi	*Saccharomyces cerevisiae*	62	◯	◯	-
	Viridiplantae	*Arabidopsis thaliana*	72	◯	◯	-
	Euglenozoa	*Trypanosoma brucei* TREU927	1	◯	◯	◯
Archaea	Euryarchaeota	*Pyrococcus furiosus* DSM 3638	2	◯	-	◯
Bacteria	Deinococcus-Thermus	*Thermus scotoductus* SA-01	0	◯	-	◯

Distributions of the Clp1 and RNA ligase family enzymes involved in pre-tRNA splicing in each representative organism are shown. Presence or absence of the corresponding gene is indicated by "◯" and "–", respectively. **Method**: To determine the presence or absence of the corresponding genes, we performed a sequence similarity search against the RefSeq database using known gene sequences, with an E-value of ≤1e−4 and query coverage of ≥30%. The query sequences (UniProt AC) were *Escherichia coli* RtcB (P46850), *Homo sapiens* RtcB (Q9Y3I0), *Saccharomyces cerevisiae* Trl1 (P09880), *Trypanosoma brucei* Trl1 (Q38AF2), and *Arabidopsis thaliana* Rnl (Q0WL81). For the eukaryotic RefSeq database, see the Dataset section in the Materials and Methods, and for the prokaryotic RefSeq database, we used the August 2018 dataset (ftp://ftp.ncbi.nlm.nih.gov/genomes/refseq/; last accessed September 17, 2019). The number of intron-containing tRNAs in each species is according to the Genomic tRNA Database (GtRNAdb) (http://gtrnadb.ucsc.edu) (Chan and Lowe 2009).

Supplementary Table S6 Nucleotide sequences of the euglenozoan *Clp1* genes optimised for *E. coli* codon usage and the amino acid sequences encoded

Tb -Clp1-t1-His Tag (Optimized nucleotide sequence)

ATGAGTGCAATCGAACAAGCGAACTTCCGCCTGCAAGCAGGCAGCGAACTGGTCGTGGTGGTGCCCTATTCAACCGAACGCTGCTCTGCCACACTGAAAGTTGTGAGTGGGGCCGGAGGCCTTG
AACCACGTGTTGATGTGCTGGGTGCGCCGGTCATTGTGGATGTCAGCTACAACCTCCCGCCGGGCATTACCTTCACCGTCTTTACCTGGAGCAACGCCAACATCCGCATTGAAGGGTCAAAACAG
CTGGTACAGAATTGCTTCCGGAGTACTACCCATCCGTTTGCGCGGTCGATTGTTGAGTACCATTGCCTGATTCACAATGCACGCCTTTTAGCGGATAAGCAGGGCCTGTTCGGTCCGATGGTGCTC
ATTTGCGGTCAGAATGATACGGAGAAGCATGCCATTTCGCGTACTCTGAGCTCCTATGCTGCGCGTACCGGCTGGGCTCCGCAGTTTGTGGATCTGGATTGTGGCATGGGTCAGCTGCTGTCCACG
CCAGGTACCGTAGCGGCCTGTGTTTCCGAATGTCCGATGACGTTAGACGAAGATACGTCGACGGGTCCGCTGTCAGTTGCGTTCTTTGTTGGTAGCACAGAACCACAGGTTAAAGGCGTTTCTGGG
GAATGGAACATGTTTGCGCCGTATGTGCACTATTGTCGCTTGCTGTTGAGTTGCGTTTCAGAACGTATTGCACGCCATAAAGGTGGCGCTGGAGGCAGCTCTGGCGCGATTATCGTGTTACCCGAGT
TGCGTGGGAGCAATGGCCTGCTCTTTGTAGTGGACATCATTCGCCAGTTCAACATCAGCCACATCTTATGCGTAGGGGACGACTTCCTGTTCTGTGGCCTGCATGAACGCATTCCACGCTTGCGCG
AACACATGGCTTCCCGTGTAGGTGGAGACATTCGTTTGGATAAACTCTCTGGCTCGCCTCACTTTCCGTCCCCTGATACTCGCACAGAACGCTTAAGCTCGATTATGGAGCGGTACTTCTTTGGTGG
CGGGGCGGTCGACTTGCAACCTAGCGAGATCAACCGCCGCTATGCGAGCATCGAGATCCTGCTGCTGAAAGAAGCCAATGGCCAAGCTGTTGTGAGTCCCGTCGAGCAAGATGCACTCGAAGGA
GTCGTGGGTTGCGTTGGCTCGTTATTTGAATCTAGTGCTATCCATGAGAAAGGCGCCCTTTCACTGGCACCGTTTGCACTTGCGCGTGTGCAGGGTATCGATGCCAATGGTGTCTCCCTGCTGGTG
AGCACCCATTCTGCCATTCCGGAACGTCTGACGATGATTGTAGGTGCGTTTCGTTGGGTCACCTCGCTCGAGCATCACCATCACCATCAC

Tb -Clp1-t1-Hisx6 (Amino acid sequence)

MSAIEQANFRLQAGSELVVVVPYSTERCSATLKVVSGAGGLEPRVDVLGAPVIVDVSYNLPPGITFTVFTWSNANIRIEGSKQLVQNCFRSTTHPFARSIVEYHCLIHNARLLADKQGLFGPMVLICGQNDT
EKHAISRTLSSYAARTGWAPQFVDLDCGMGQLLSTPGTVAACVSECPMTLDEDTSTGPLSVAFFVGSTEPQVKGVSGEWNMFAPYVHYCRLLLSCVSERIARHKGGAGGSSGAIIVLPELRGSNGLLFVV
DIIRQFNISHILCVGDDFLFCGLHERIPRLREHMASRVGGDIRLDKLSGSPHFPSPDTRTERLSSIMERYFFGGGAVDLQPSEINRRYASIEILLLKEANGQAVVSPVEQDALEGVVGCVGSLFESSAIHEKG
ALSLAPFALARVQGIDANGVSLLVSTHSAIPERLTMIVGAFRWVTSLELEHHHHHH

Tb -Clp1-t2-His Tag (Optimized nucleotide sequence)

ATGAGTTCAAACTGCCGCACGGAAGAGTTCAATTTGGAGCGCAAAGAACTGAAAATTCGCTTCCGCTCTTCCGGCTATGTCGTACTCACCGATGGGGCGGCGACGATCTTTGGTGCTCCACTGAAG
AAGAACACTCGTTACGACTTTTCAATGTGCTCAATCCCAGTGGTTTCTCCGGTTGCGTGTCGGTTGCATGTTGGCGGAGACTTCACTTCGGTGGTGACGTACATTCGCACAGACGTGTATGATATCC
ATGCAGTCCTGGACTTCGCTCGCCATGAGGCGAGCAAACGTGGTGTGCCCAGCCTGAACGACACGAATCCTGGCGGAACAAGCAGCAATAGCCTGAAAGAAGGCCCGTGGGGTCCGCGTGTGC
TGGTAGTTGGCGATGTTAATACGGGCAAAAGCTCCCTGTGCCGCAGTCTGGCCAACATGGCGGTGGCCTCGCAAGTGCACGGTGTTGCACTGGTTGATGTAGATGTTGGTCAGCAGGGGATTACG
TGTCCGGGTTCCGTGGCAACAGCTTTTGTGGACAACTATCTCCCCATTGATGAGGGCTTTAACACCGTAATGCCCTTAACCGCCTTCTTTGGCGACAAAACCGTTAATGCCTCTACTCGTGGGCGG
TATCTGGACCTCTGTGCGAGCTTAGCACGTGGCATTATCTCGTTTTCCTTGGCAACTCCGAAATTTGCGGCTGGTGGCGTAATTGTCAACACGATGGGTTGGGTCACCGATATGGGCCTGGATCTGC
TTTTCCAGCTGCTGAGCGTCTTTTCGATTACGCATGTCGTCGTTTGTGGCAGTGGAAATAAGCTGACAGAAACCTTACGCAATGCGGTGATTGACGAGAAAATTATCTTCCTGAAGTATCCGAAACAA
ACCGGGGTCTTTAAACGCAAAGGTAACGTGCGCGATTCTTGGCGTGCGGAACAGATCGTGTCCTACTTCCAAGGGACCAAACGTACCCCTTTACTGTCATATCGTGCCGTGTGCTACGTTAAGGAT
GTACACTTTATCCACGCCTTGAAACTGGAACCGTTAAGCTGGAAAGATGTGGAACCGCTTTCGCTGGCCGCTGTGAGTTGGACCGATAGCCTGGAAGCGGTGAATGATATTAACGTTGCAGGCTTT
ATCGTGTTACTCGAAGTCGGTGAAACCTTCTTTTCGTTTCTGAGTCCAGTTGCGGGTACTCTGCCTAAACCGTTCATTCTTGTATCTCCGACCATTCGCCTTCCTCGCGATAAAGTCCCGCCATTGCA
GGCCCCGCTCGAGCATCACCATCACCATCAC

Tb -Clp1-t2-Hisx6 (Amino acid sequence)

MSSNCRTEEFNLERKELKIRFRSSGYVVLTDGAATIFGAPLKKNTRYDFSMCSIPVVSPVACRLHVGGDFTSVVTYIRTDVYDIHAVLDFARHEASKRGVPSLNDTNPGGTSSNSLKEGPWGPRVLVVGD
VNTGKSSLCRSLANMAVASQVHGVALVDVDVGQQGITCPGSVATAFVDNYLPIDEGFNTVMPLTAFFGDKTVNASTRGRYLDLCASLARGIISFSLATPKFAAGGVIVNTMGWVTDMGLDLLFQLLSVFSIT
HVVVCGSGNKLTETLRNAVIDEKIIFLKYPKQTGVFKRKGNVRDSWRAEQIVSYFQGTKRTPLLSYRAVCYVKDVHFIHALKLEPLSWKDVEPLSLAAVSWTDSLEAVNDINVAGFIVLLEVGETFFSFLSPV
AGTLPKPFILVSPTIRLPRDKVPPLQAPLEHHHHHH

Tb -Clp1-t3-His Tag (Optimized nucleotide sequence)

ATGGCGAATACCGCGCTGCTGGAGCGCGAATATGCGCTGCCGCCGATGGGTGAACTGTGCTTGGCCACGGCCTCTAGCGGTGGCGCGGCTACCGTGACGTTACTGGCTCAAGTTGAGGACGGC
GGGGAAGAACCTCGCGCAGAGATTTTCGGCACGGAACTCTCCACCGGTGTGGTGGTTCATCTTCCTGTCGCTCGCTCGCTGGCGGTGTTCTCCCCAACAGGTTGTCGCTTAGTGCTCACCGCGAG
TTCAGCCGTTCACCAGATCTGCTATGGTACCACATGCAATGCCACGCGGGCACGCTCGGTAGCGGACATTCACACGCATCTGGAAGTTCAGCGTGTGAAAGCGCGTCGTACCGGCGCAGATGGT
ATTGGCCCGCATGTGCTGTTTGTCGCGGAACGTCGCGCAGTGGGTACAAGCACCTACGTACGCACTCTGATCAACTATGCGGTTCGCCTGGGGTACCATCCGCTGCTTTTGGATGCCTCCGTTGA
AGCGCCACGTTTTGGCTATCCTGGCGTCGTGTCCTTGTATGCAATGCAGTACACCATTGACATTGAGAACGAAATGGCGTTTGTCCCGGGATTACACAGCCATCAAGGGACGAAGAAACATGAAGA
TCCAGCGTTATTCCTGCACATCCTTCGTCAGATGATGCGGCTGTCAACCGAACGCATGGCTCGCTCGGATCGCTGCCGCGTTGGCGGCATTTTCGTAGATTATGGCACCATTAGTCGTGCAGTGGT
CGAGGACGCTGAAGCCTGGGAATGTGCGGAAGAGAAACCCGAAGGTCGCCCGAAAGTTAACCCGCTGGACGTGTTGGTGTCTACGATTTTGGCGGCCGGCATTGACCATGTGTTTGTGGTTGGTA
GTTCATGGCTGCGCTTCAAGATTGCGCAGCGTTTACACCAGGAGTCAGGCGCACAGTCTGAAATCCCGCAAATTACTCCGAGTACCATCACCTGTTCTAACGGCTTGAAAGTTCAGTTGTTCCTGTT
AGATAGCACCGAATGTGGTGCAGTTCCGGATGATGCCTTCTTCAACCGCCAATGCTGGCTGCAGTACTTCTTTGGTTCGCGTACTATGGCTGTCAAACCAACCCTCTTTACCGTGGATGCCAGCCT
TATTCGGCTGGTCACTATTGGGCGTGGTGATACGTCCGGAACAAGCACGTTTATGCCCATGATCGACGATGATTCTGACCACCAAGATCCCACGGTAAGCGGACAGGCTGATGTAGCCCTGACTTA
CGTCCATCCGCAGGATGTGGATATCAAGAATCGTGTACTGGCCCTGAGCACAGCGACCGAACAGGAAGAACTGCCGGATGGGACTTTACAACGCATCCCTTTTGCAGTGTTTGAAAGCCGTCTGA
AACGTGGGCTTCTGATGGGTTTTGCGCTCGTCGAGTCGGTCACAGCAGGCAGTGTGACCCTGCTGACGAATGCTGCAGGAATCCGCAAAGATATTGGCCTCTGCTTTATCGTTACCGACCAGCAG
CTGATGGCCCAAGCCGACGTAGAGCCACCGACTCTCGAGCATCACCATCACCATCAC

Tb -Clp1-t3-Hisx6 (Amino acid sequence)

MANTALLEREYALPPMGELCLATASSGGAATVTLLAQVEDGGEEPRAEIFGTELSTGVVVHLPVARSLAVFSPTGCRLVLTASSAVHQICYGTTCNATRARSVADIHTHLEVQRVKARRTGADGIGPHVLF
VAERRAVGTSTYVRTLINYAVRLGYHPLLLDASVEAPRFGYPGVVSLYAMQYTIDIENEMAFVPGLHSHQGTKKHEDPALFLHILRQMMRLSTERMARSDRCRVGGIFVDYGTISRAVVEDAEAWECAEE
KPEGRPKVNPLDVLVSTILAAGIDHVFVVGSSWLRFKIAQRLHQESGAQSEIPQITPSTITCSNGLKVQLFLLDSTECGAVPDDAFFNRQCWLQYFFGSRTMAVKPTLFTVDASLIRLVTIGRGDTSGTSTFM
PMIDDDSDHQDPTVSGQADVALTYVHPQDVDIKNRVLALSTATEQEELPDGTLQRIPFAVFESRLKRGLLMGFALVESVTAGSVTLLTNAAGIRKDIGLCFIVTDQQLMAQADVEPPTLEHHHHHH

Li -Clp1-t2-His Tag (Optimized nucleotide sequence)

ATGCAGCCCCGCAAAACGCATACGAGCCTTCACTTGCGGCAAGAAGCGGTGACCATCCAGTGGAATGCGGGTCAGGAAGGCGGCAGTGTTCTGCTGTTATCAGGCAAGGTCGAGTTATTCCGCA
GTAGCCTGACCCGTAATCTGCGCTATGCGTTTCCAGCGGAAGCGTGCATTGTCCTGGAGGCCTTTGGTGACGCCGTAGTGCAAATTGATGGCGATGCAATCACAGTCCAGACTCCCATTTCAGGTG
CGCTCGATGAAATCCACGCCCTGCTGGATACCGCACGTGTTGACGCTATGCTGGCGATTGACGAACGCTCTAAGAAATCCCTTTCGAGCTCTGAAGAGCTGAAAGACTCTTGGCAAGGGCCACGG
GTCCTCGTTGTTGGTGAGAATCAGTGGGAACGTGAAGTCGTGAGTCGTGCCTTACTGAACCTTGCGGTTCGTCATGGCAGCCCGTATGGCATTTGCTACGTAGACGTGGATGTTGCCATGCCTATG
GTGGGTTGTCCAGGAACCGTGTCAGCCGCTTTCGTGGAGGAACCTGTGACAGCGCCGGAAGATTTCAGCGTCATGATGCCGCTGACCTTCTTTCATGGCGCAGCATCGGTAACGAGCGCCACCC
GCAAACGCTATCTGGATCTGTGCGTTTGTGCAGCGCAAGCGGCAACCTCTTTGGGTTTTGCCAACTCCAAATTTGAAGCAGGCGGGTTTCTGATCCACTCGCTGTCTCCGAGTACGGATATTCAGC
ATGACGTGCTGTCCGACGTGATTAGCATCTTCGCCGTAACACACGTTGTTGTCACCGGGGCAGATTGGGAACTCGAAAAGTTTCTCAACAATGCGGTCGTTGGCCGCACTGTGCACTTCGTGCGTT
TGCCGAAATTAGCCGGTGGTCAAAGCCCGAGCGCTGCCGGTGCTGAACAGCGCCGTCGCGCTCAGCTGGAGCATTACTTCTTCGGTACGCCGCGTACTCCACTGATGCCCGTTCGCGGGGTGG
CACGCATGTCGGAACTTGTACTGTTGCATGCGGAAACGTTTGAACCGCTCTCGTGGCGTGAAGTGCCGGATTTAGGCTTAGCAGCTGTCGTTTGGGCGGATACTGCTGCCAGTGCTGTCGAGGCG
AACGTTGCGGGCTTTGTAGCCTTGCTGGAGGTCGGCAAACAGTTCGTGTCCTTTCTGGCGCCTTCCGGAGGAGAACTGCCGAAACCGTTTCTGGTGGTAAGCCCGTCACTGCATCTTCCGCGCGA
GTTGGTGATGCCACTGCCTGTGACCCTCGAGCATCACCATCACCATCAC

Li -Clp1-t2-Hisx6 (Amino acid sequence)

MQPRKTHTSLHLRQEAVTIQWNAGQEGGSVLLLSGKVELFRSSLTRNLRYAFPAEACIVLEAFGDAVVQIDGDAITVQTPISGALDEIHALLDTARVDAMLAIDERSKKSLSSSEELKDSWQGPRVLVVGEN
QWEREVVSRALLNLAVRHGSPYGICYVDVDVAMPMVGCPGTVSAAFVEEPVTAPEDFSVMMPLTFFHGAASVTSATRKRYLDLCVCAAQAATSLGFANSKFEAGGFLIHSLSPSTDIQHDVLSDVISIFAV
THVVVTGADWELEKFLNNAVVGRTVHFVRLPKLAGGQSPSAAGAEQRRRAQLEHYFFGTPRTPLMPVRGVARMSELVLLHAETFEPLSWREVPDLGLAAVVWADTAASAVEANVAGFVALLEVGKQFV
SFLAPSGGELPKPFLVVSPSLHLPRELVMPLPVTLEHHHHHH

Supplementary Table S7 Oligonucleotide sequences used for the phosphorylation assay

Oligonucleotide name	RNA/DNA	Oligonucleotide Sequence	Length (nt)
R20-FAM	RNA	5' - UAAUACGACUCACUAUAGGG -3' (FAM)	20
R20-comp	RNA	5' - CCCUAUAGUGAGUCGUAUUA -3'	20
D20-FAM	DNA	5' - TAATACGACTCACTATAGGG -3' (FAM)	20
D20-comp	DNA	5' - CCCTATAGTGAGTCGTATTA -3'	20

The sequence of oligonucleotide R20-comp is complementary to that of the R20-FAM oligonucleotide. This is also true for the D20-comp and D20-FAM oligonucleotides. FAM, carboxyfluorescein.

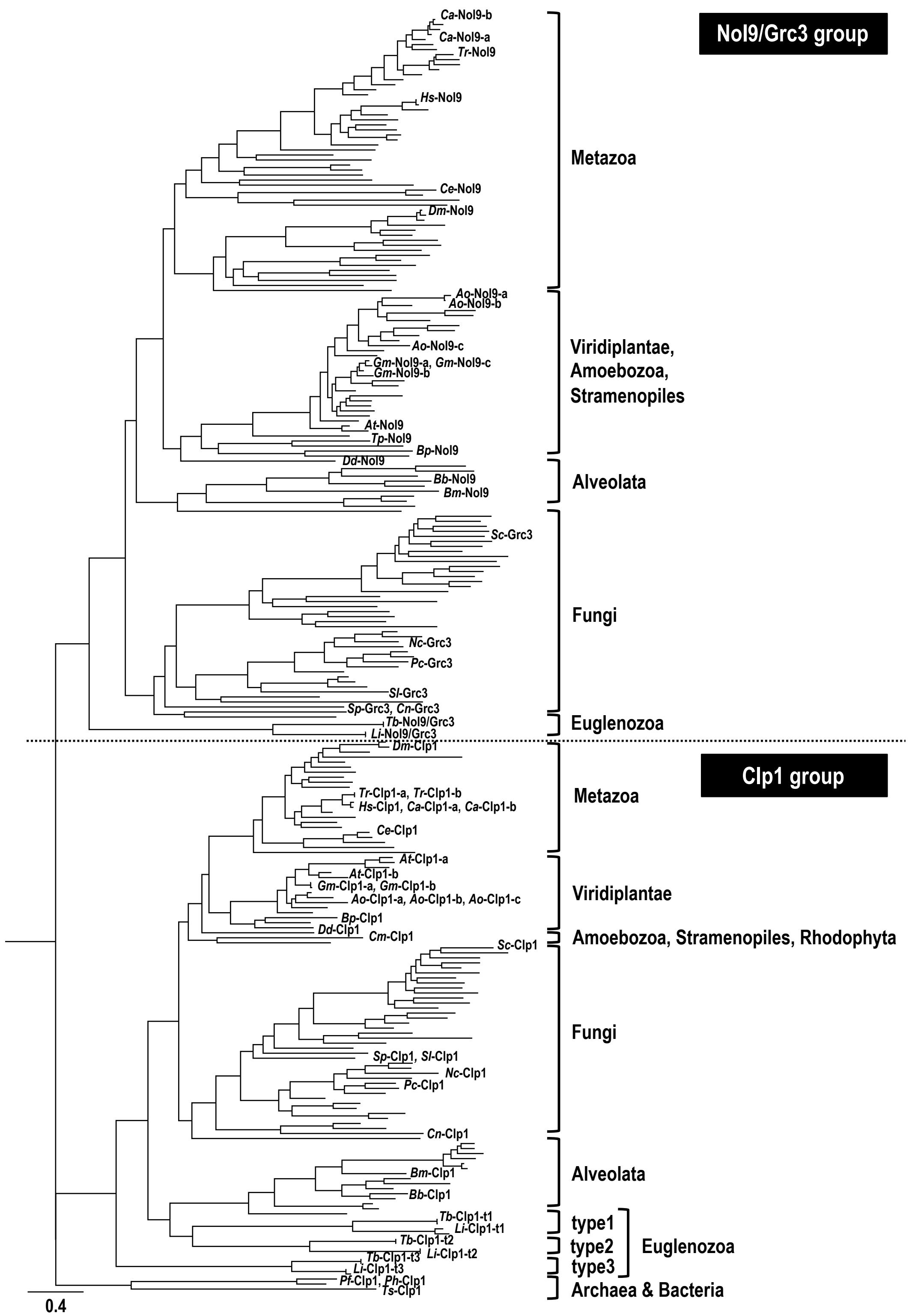

Supplementary Fig. S1 Classification of Clp1 family proteins (both Clp1 group and Nol9/Grc3 group) analyzed in this study. A phylogenetic tree based on 254 Clp1 family proteins (both Clp1 and Nol9/Grc3 groups) consisting of 251 protein sequences from 154 eukaryotes, two protein sequences from two archaea, and one protein sequence from a bacterium is shown (see also Supplementary Table S2b). The phylogenetic tree was constructed from their full-length amino acid sequences. The LG+F+R8 model was used for this phylogenetic tree. Midpoint rooting was applied during tree visualisation. The scale bar under the tree indicates the number of amino acid substitutions per site. The protein names of representative species and taxonomic groups (Kingdom to Phylum) are listed next to the phylogenetic tree. Prokaryota, Archaea, and Bacteria are described as domains. Types 1–3 are protein types of Euglenozoa Clp1 group proteins classified according to their sequence similarities. Horizontal dotted lines were used to divide the Clp1 and Nol9/Grc3 groups.

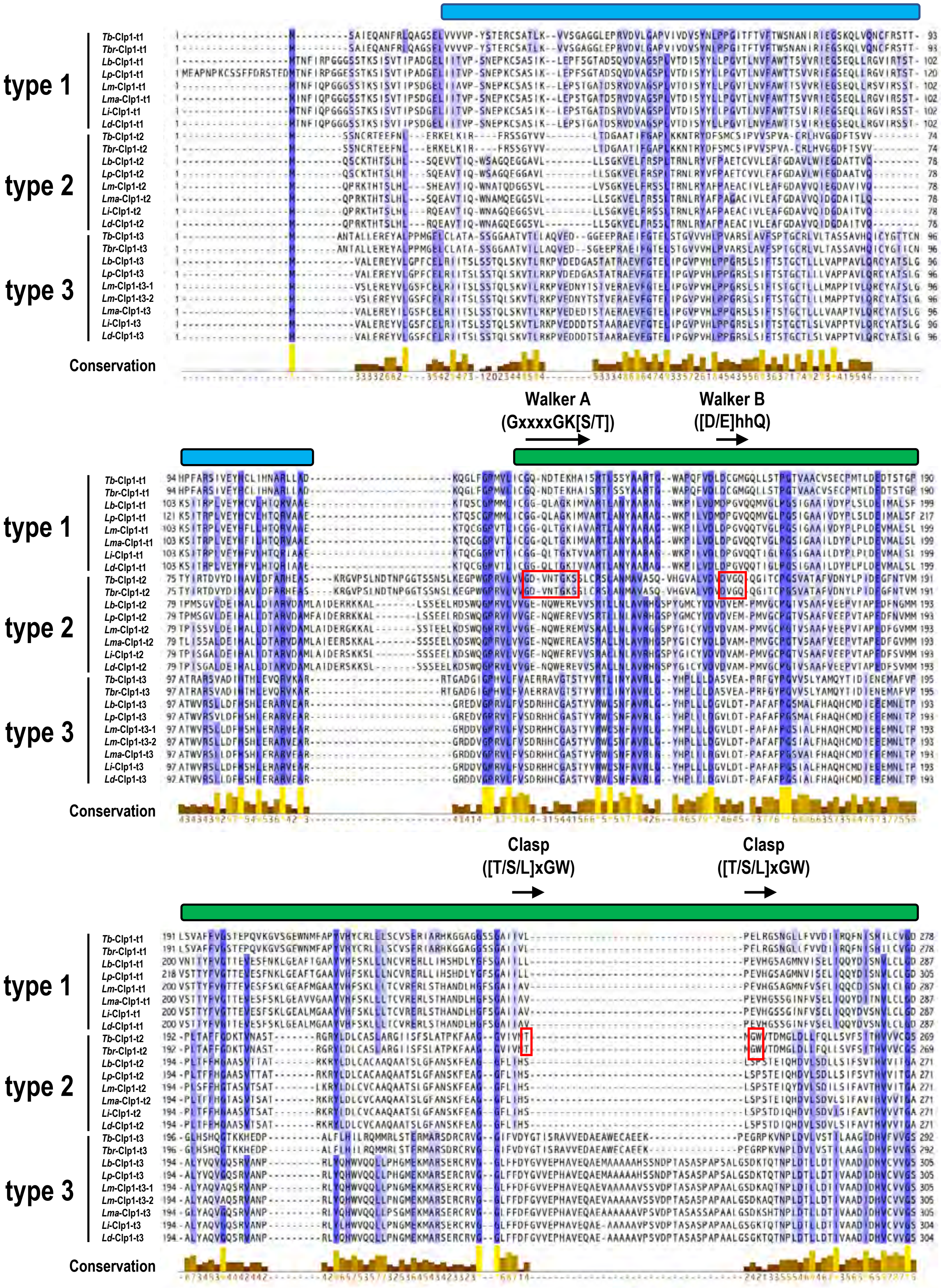

Supplementary Fig. S2

The legend for this figure is placed on the next page.

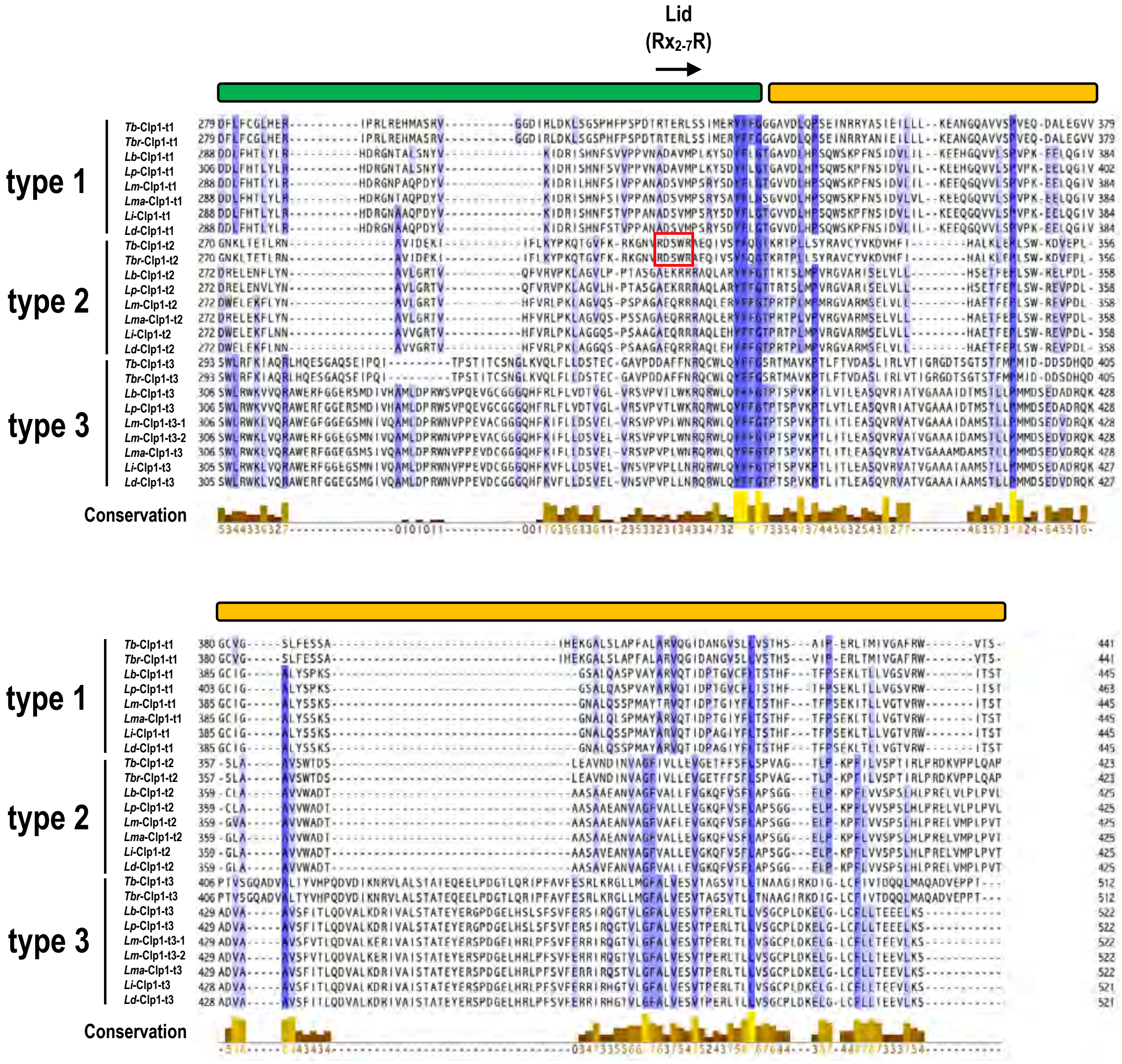

Supplementary Fig. S2 Amino acid sequence alignment of Euglenozoa Clp1 group proteins classified as types 1–3. A total of 25 (from eight species; Supplementary Table S3) of 33 Euglenozoa Clp1 family proteins (Supplementary Table S2a), excluding the Nol9/Grc3 group proteins, were used for the sequence alignment. Rectangular boxes above the sequences indicate their protein domain structures (see Supplementary Table S4): Clp1_eN1 (light blue), Clp1_P (green), Clp1_euC1 or Clp1_euC2 or Clp1_euC3 (yellow). The motif name (top), the active site consensus sequence (middle), and its region (arrow at the bottom) are shown for each sequence. The lower part of the figure shows the conservation scores of the alignment. Red boxes indicate areas in which the sequence of the active site is completely conserved. See Supplementary Table S3 for species and protein information used for the analysis.

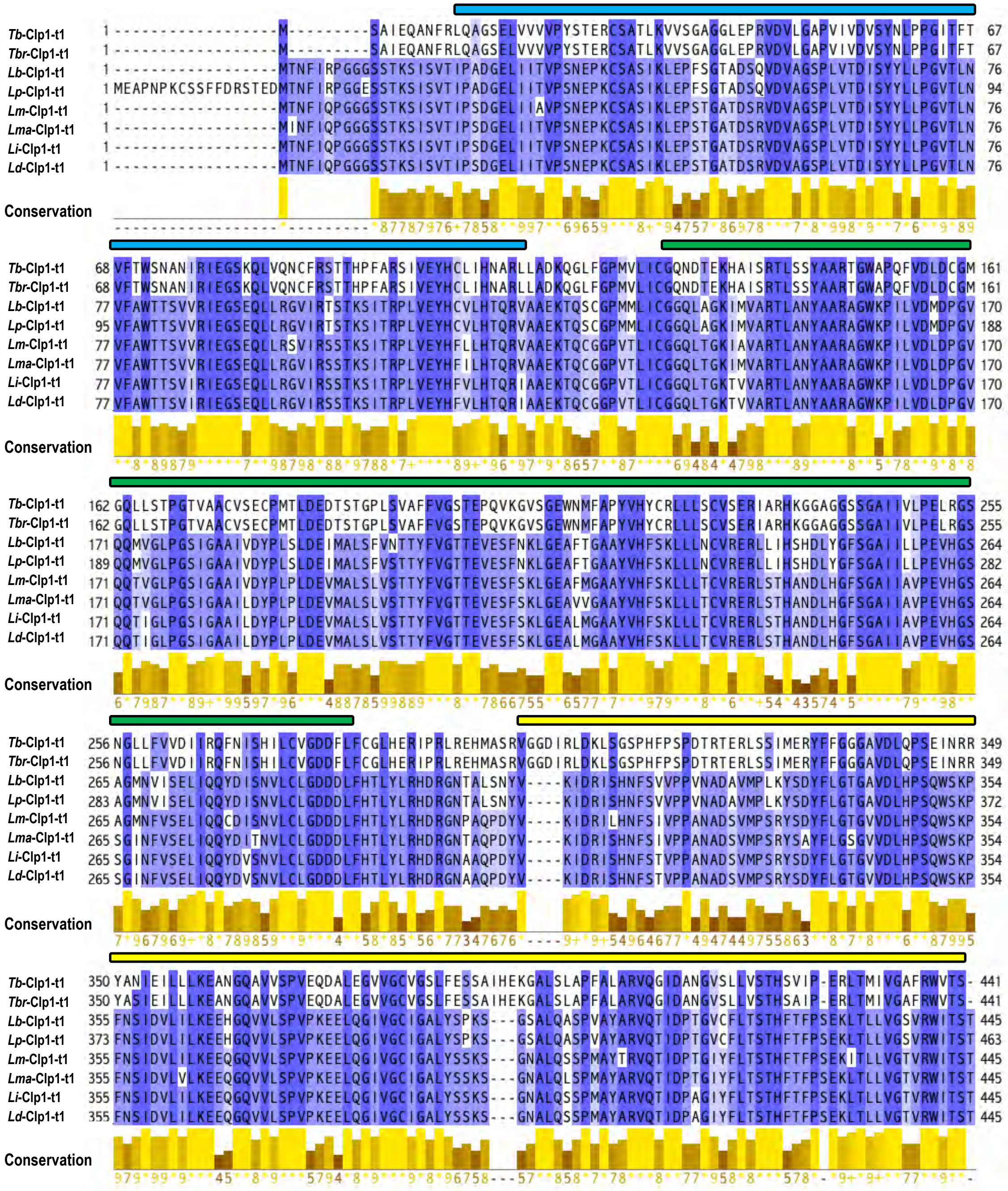

Supplementary Fig. S3 Amino acid sequence alignment of the Euglenozoa Clp1 group proteins (type 1). Rectangular boxes above the sequences indicate protein domain structures (Supplementary Table S4): Clp1_eN1 (light blue), Clp1_P (green), and Clp1_euC1 (yellow). The lower part of the figure shows the conservation scores of the alignment. See Supplementary Table S3 for species and protein information used for the analysis.

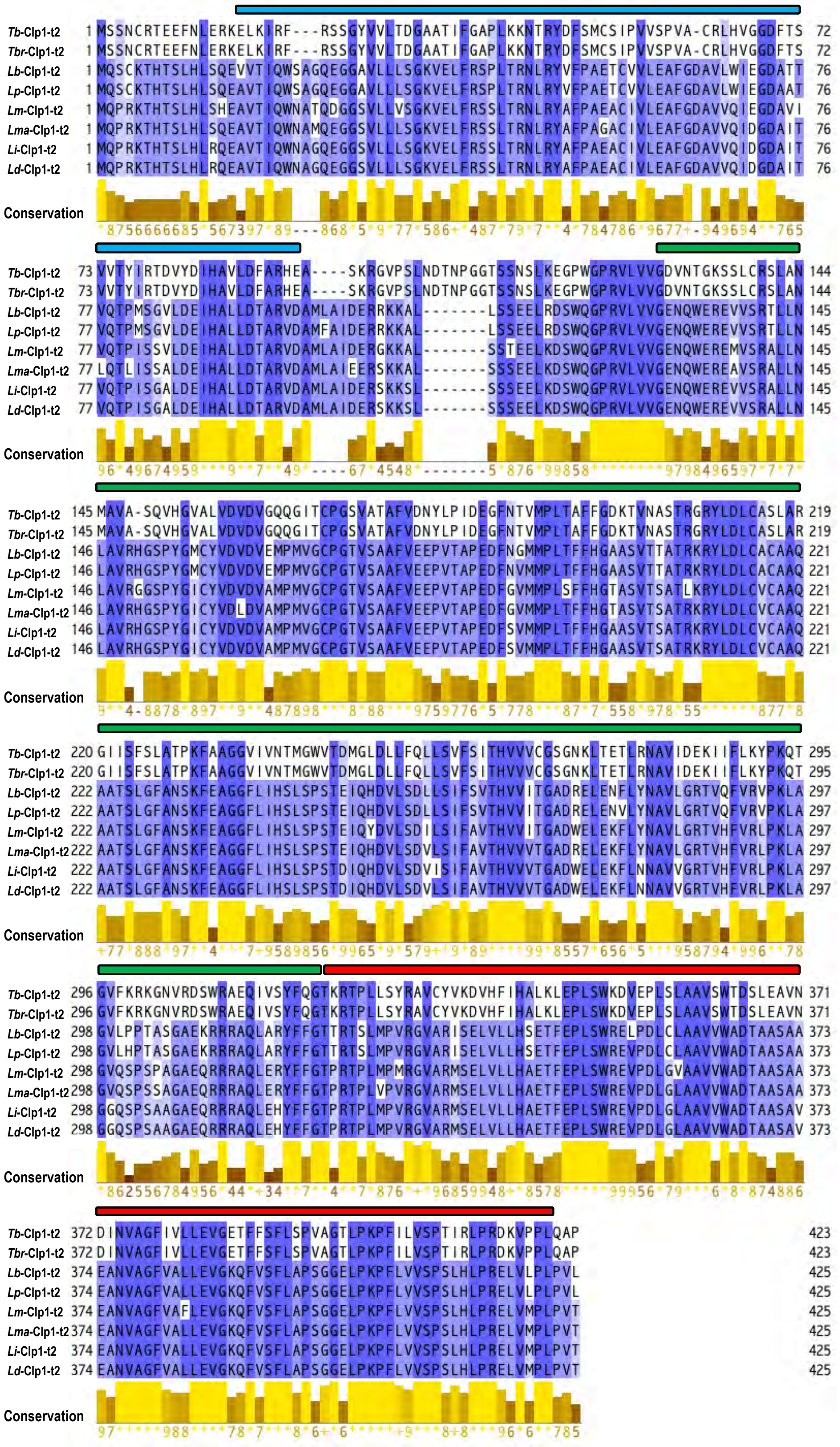

Supplementary Fig. S4 Amino acid sequence alignment of the Euglenozoa Clp1 group proteins (type 2). Rectangular boxes above the sequences indicate protein domain structures (Supplementary Table S4): Clp1_eN1 (light blue), Clp1_P (green), and Clp1_euC2 (red). The lower part of the figure shows the conservation scores of the alignment. See Supplementary Table S3 for species and protein information used for the analysis.

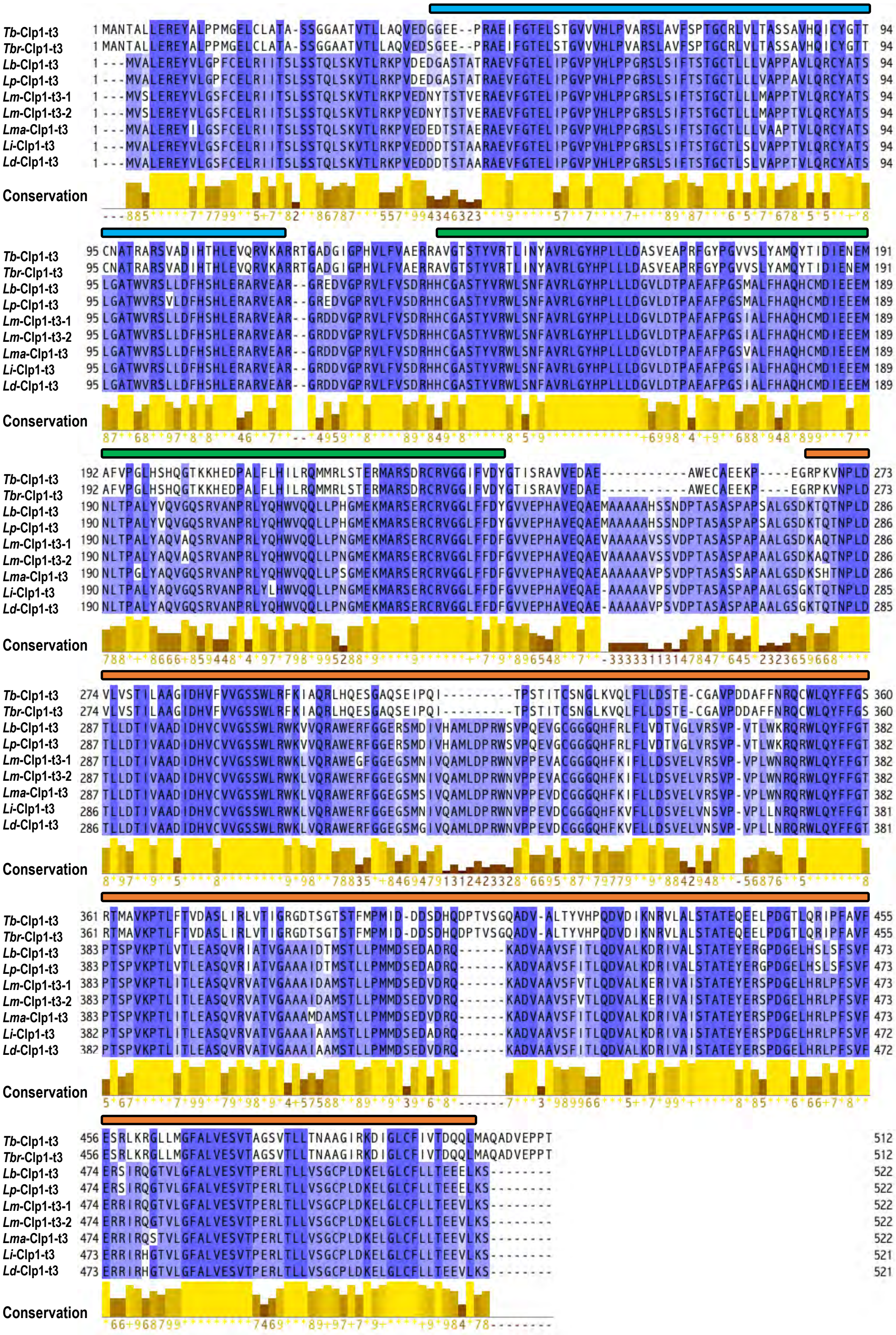

Supplementary Fig. S5 Amino acid sequence alignment of the Euglenozoa Clp1 group proteins (type 3). Rectangular boxes above the sequences indicate protein domain structures (Supplementary Table S4): Clp1_eN1 (light blue), Clp1_P (green), and Clp1_euC3 (orange). The lower part of the figure shows the conservation scores of the alignment. See Supplementary Table S3 for species and protein information used for the analysis.

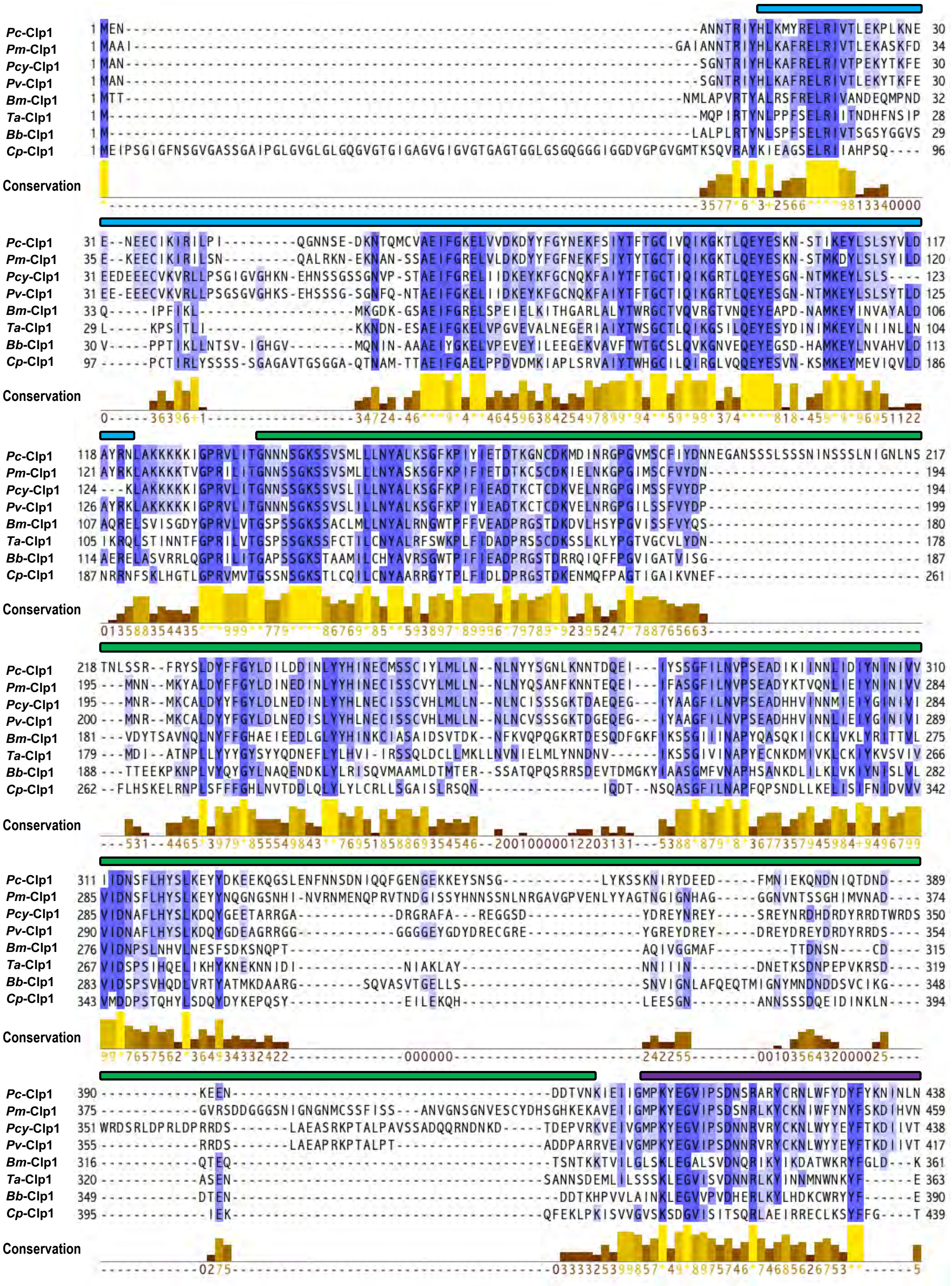

Supplementary Fig. S6

The legend for this figure is placed on the next page.

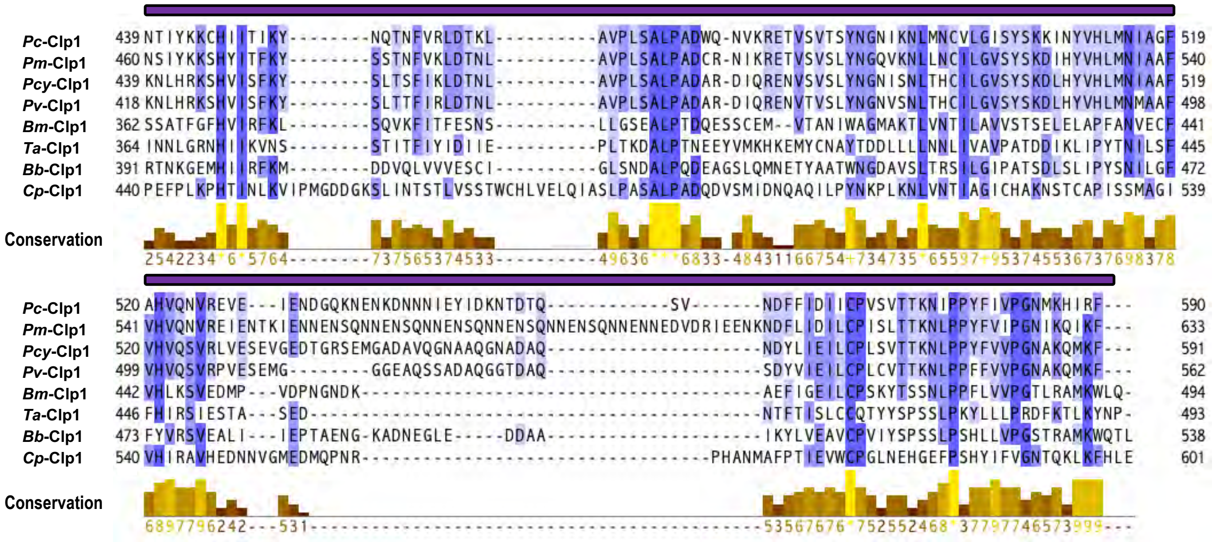

Supplementary Fig. S6 Amino acid sequence alignment of the Alveolata Clp1 group proteins. Eight samples of Alveolata Clp1 group proteins (Supplementary Table S3) were randomly selected from 15 samples of Alveolata Clp1 group proteins (Supplementary Table S2b). Rectangular boxes above the sequences indicate protein domain structures (Supplementary Table S4): Clp1_eN1 (light blue), Clp1_P (green), and Clp1_alC (purple). The lower part of the figure shows the conservation scores of the alignment. See Supplementary Table S3 for species and protein information used for the analysis.

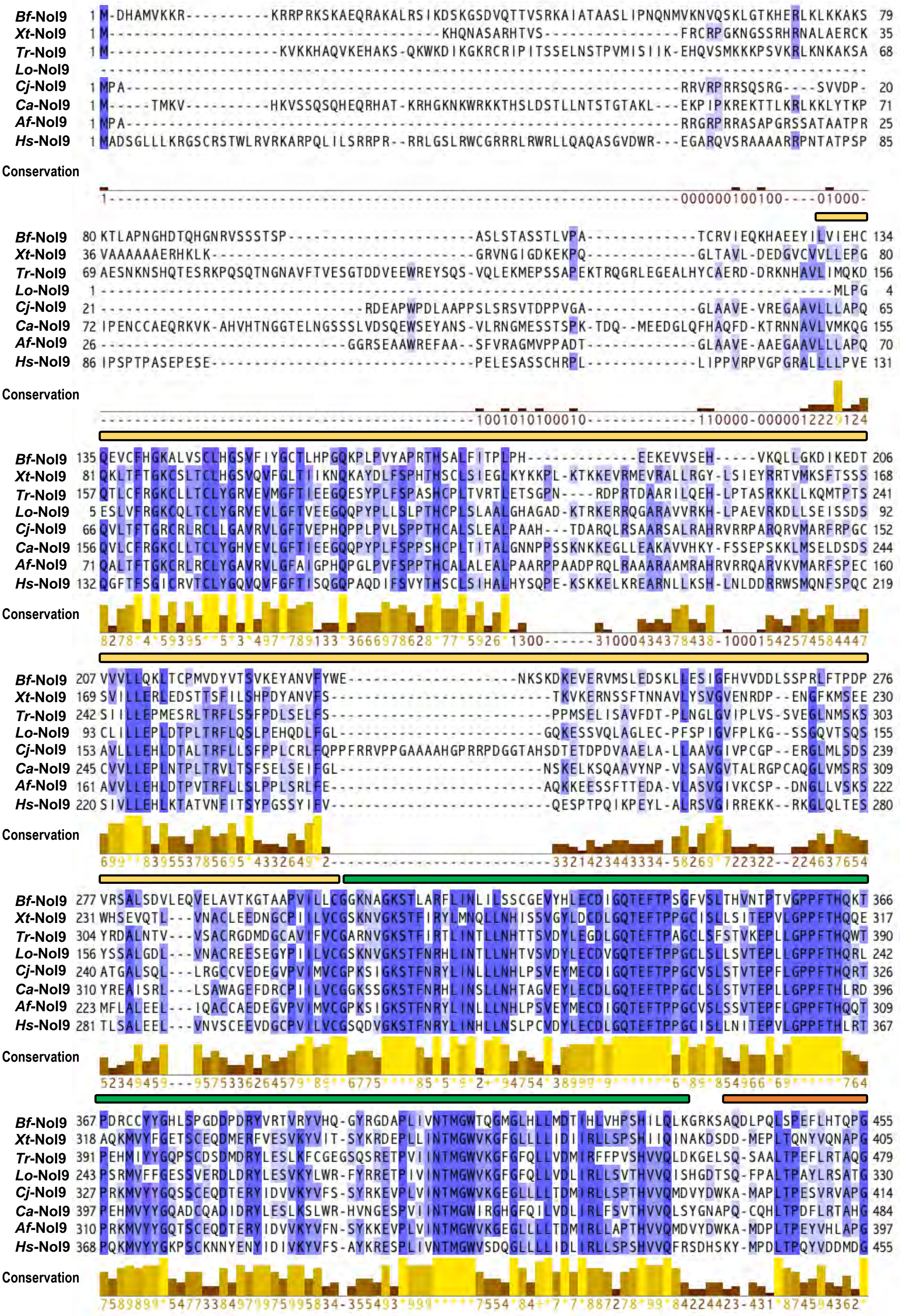

Supplementary Fig. S7

The legend for this figure is placed on the next page.

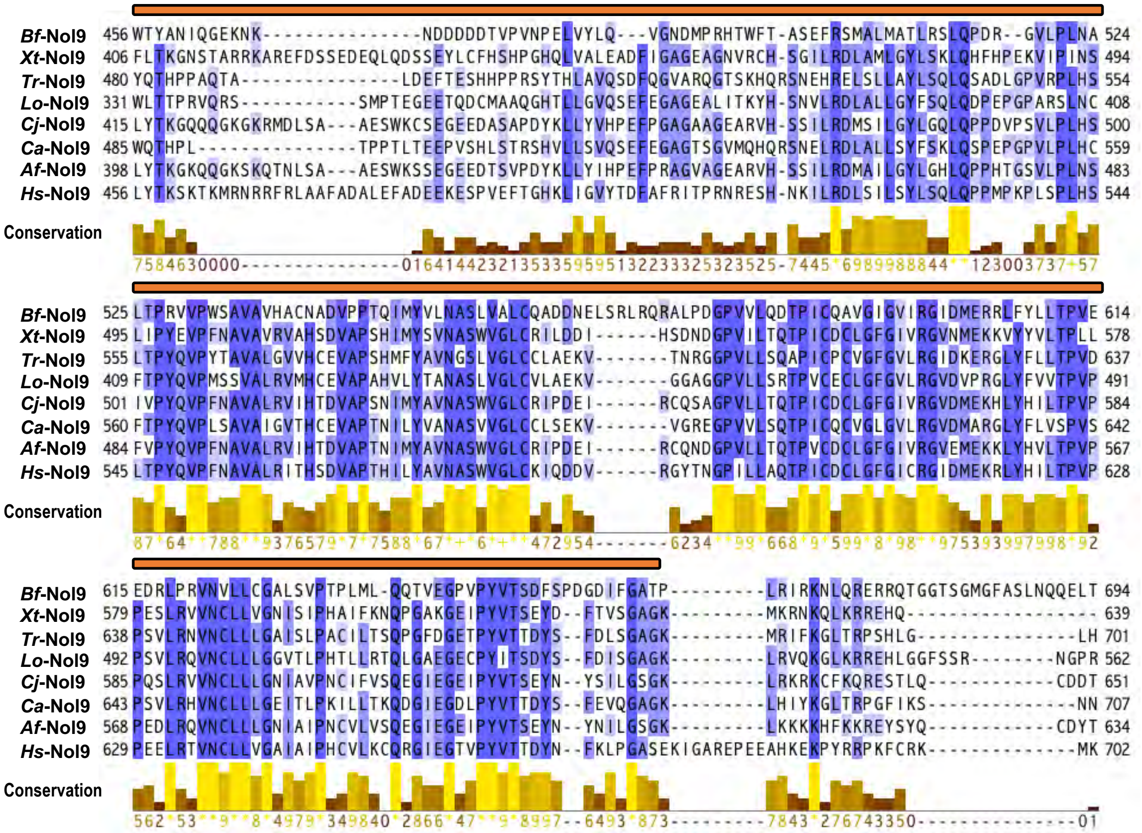

Supplementary Fig. S7 Amino acid alignment of the Metazoa Nol9/Grc3 group proteins with the Nol9_eN1 domain. Eight Nol9/Grc3 group proteins (Supplementary Table S3) from 28 Metazoa sequences containing the Nol9_eN1 domain (Figure 3) were used. Rectangular boxes above the sequences indicate protein domain structures (Supplementary Table S4): Nol9_eN1 (light yellow), Clp1_P (green), and Nol9_eC1 (orange). The lower part of the figure shows the conservation scores of the alignment. See Supplementary Table S3 for species and protein information used for the analysis.

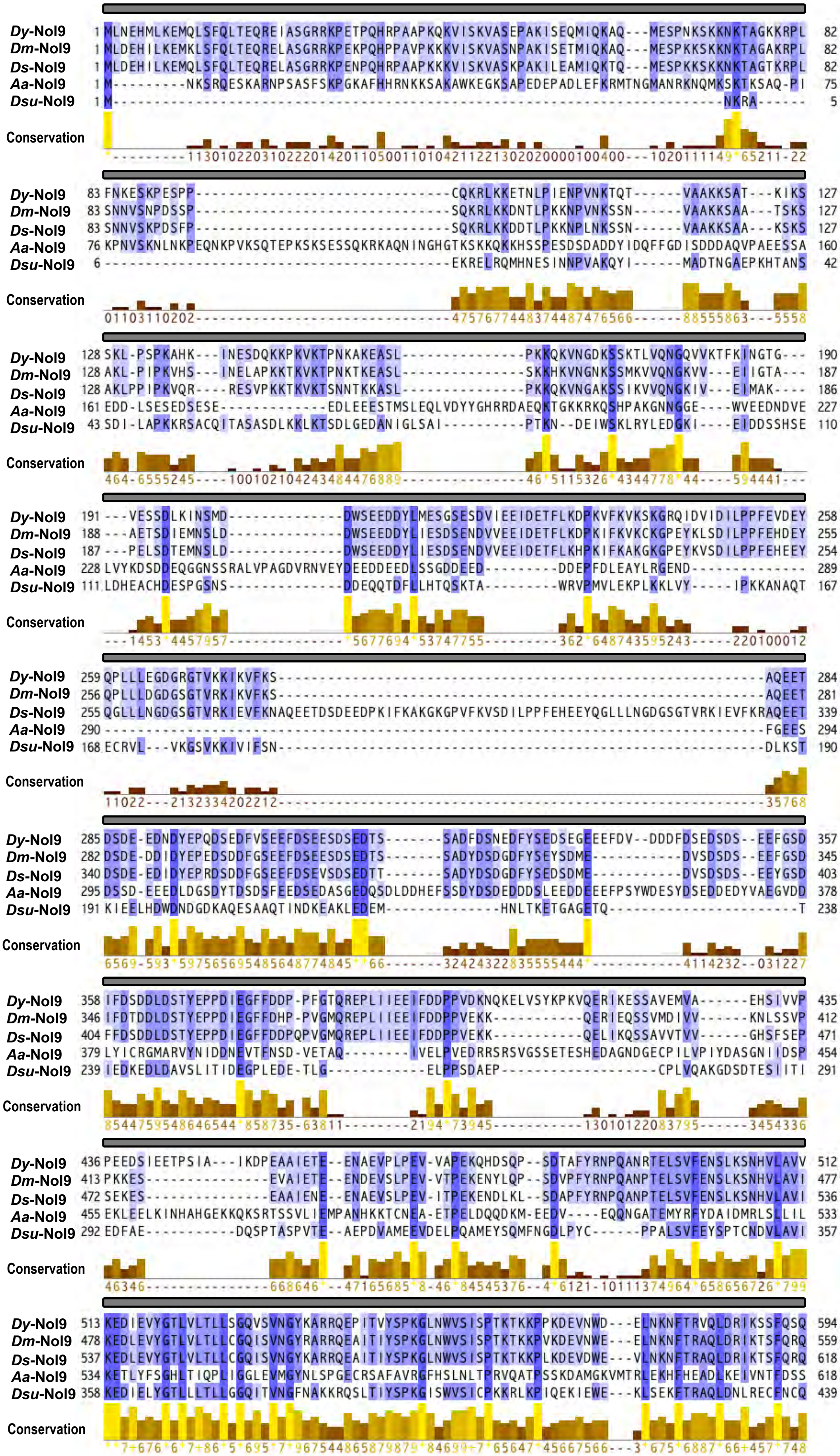

Supplementary Fig. S8

The legend for this figure is placed on the next page.

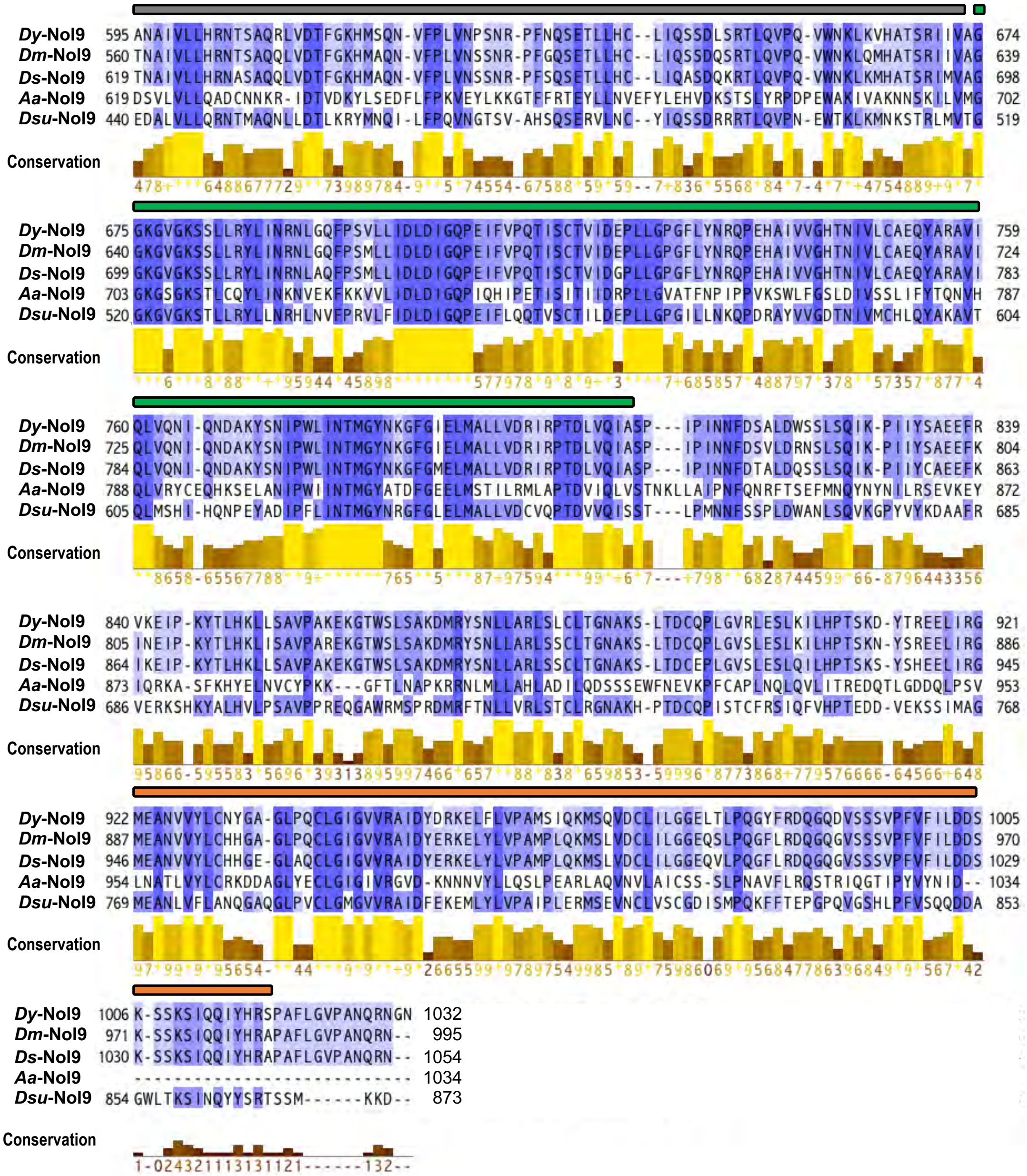

Supplementary Fig. S8 Amino acid alignment of the Metazoa Nol9/Grc3 group proteins with the Nol9_eN2 domain. Five Nol9/Grc3 group proteins containing Nol9_eN2 domain were used (Supplementary Table S3). Rectangular boxes above the sequences indicate protein domain structures (Supplementary Table S4): Nol9_eN2 (grey), Clp1_P (green), and Nol9_eC1 (orange). The lower part of the figure shows the conservation scores of the alignment. See Supplementary Table S3 for species and protein information used for the analysis.

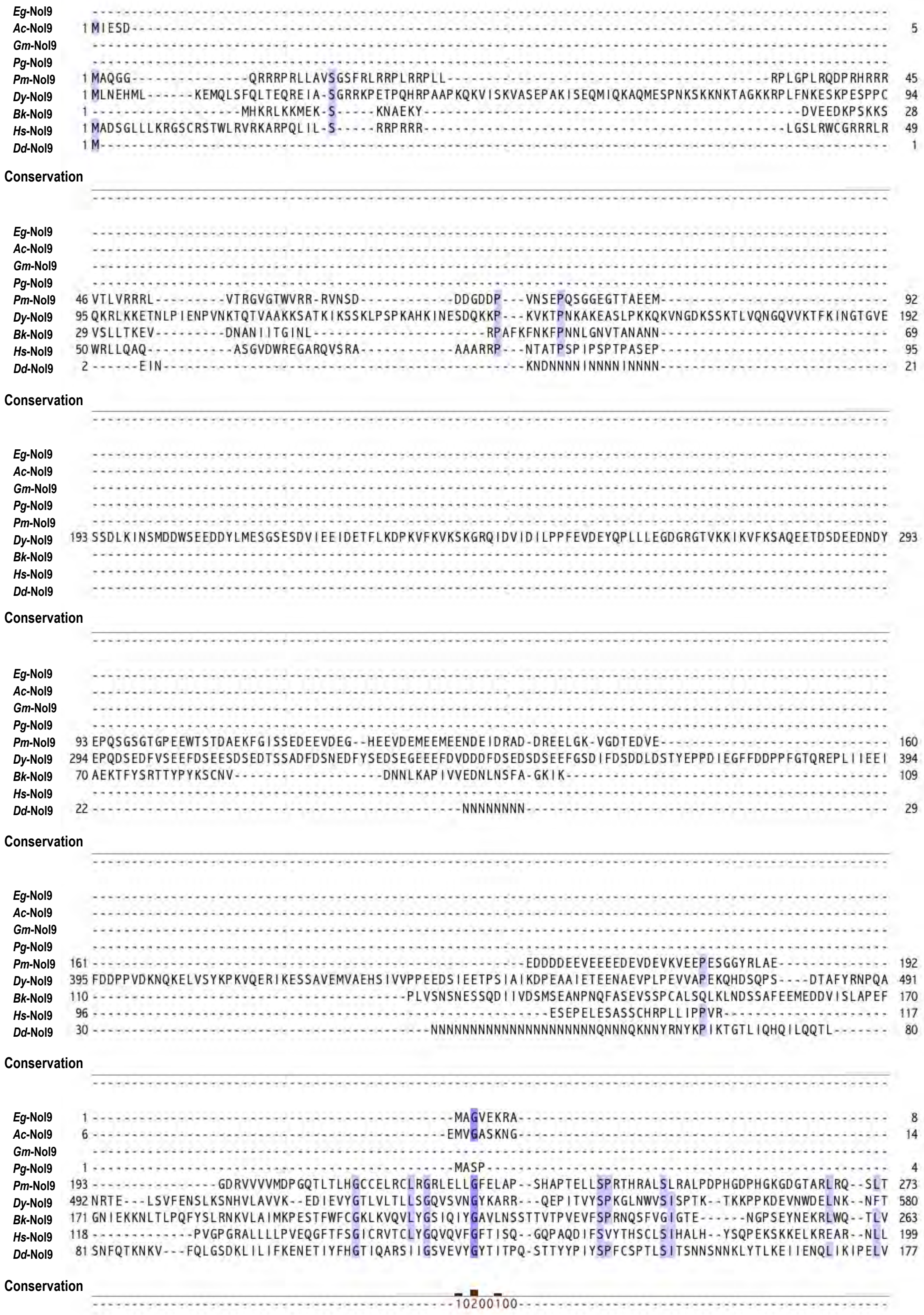

Supplementary Fig. S9

The legend for this figure is placed on the next page.

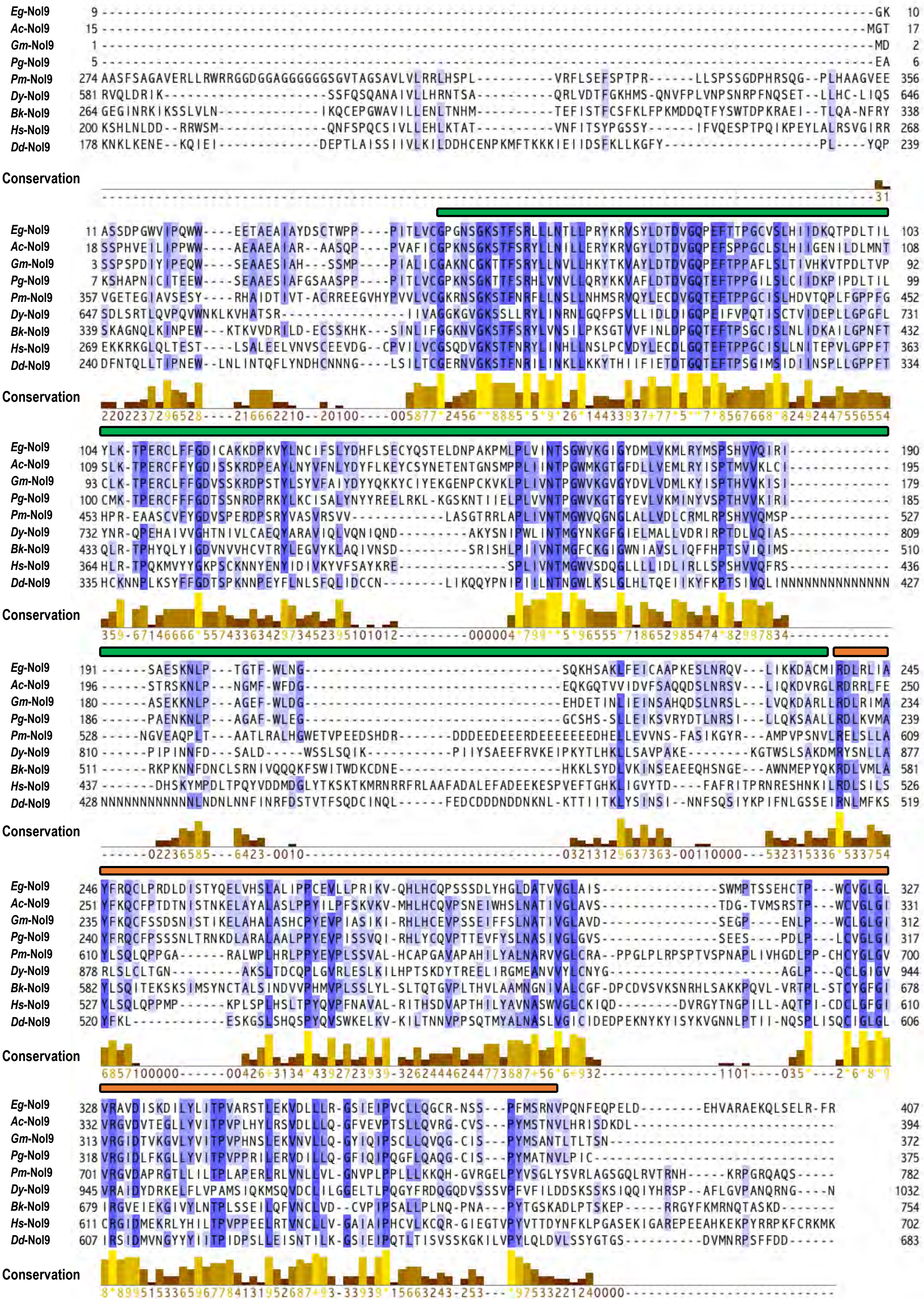

Supplementary Fig. S9 Amino acid sequence alignment of Nol9/Grc3 group proteins with the Nol9_eC1 domain from Metazoa, Viridiplantae, and Amoebozoa. Nine examples of Nol9/Grc3 group proteins (Supplementary Table S3) were randomly selected from 55 proteins of Metazoa, 31 proteins of Viridiplantae, and one protein of Amoebozoa (Supplementary Table S2b). Rectangular boxes above the sequences indicate protein domain structures (Supplementary Table S4): Clp1_P (green) and Nol9_eC1 (orange). The lower part of the figure shows the conservation scores of the alignment. See Supplementary Table S3 for species and protein information used for the analysis.

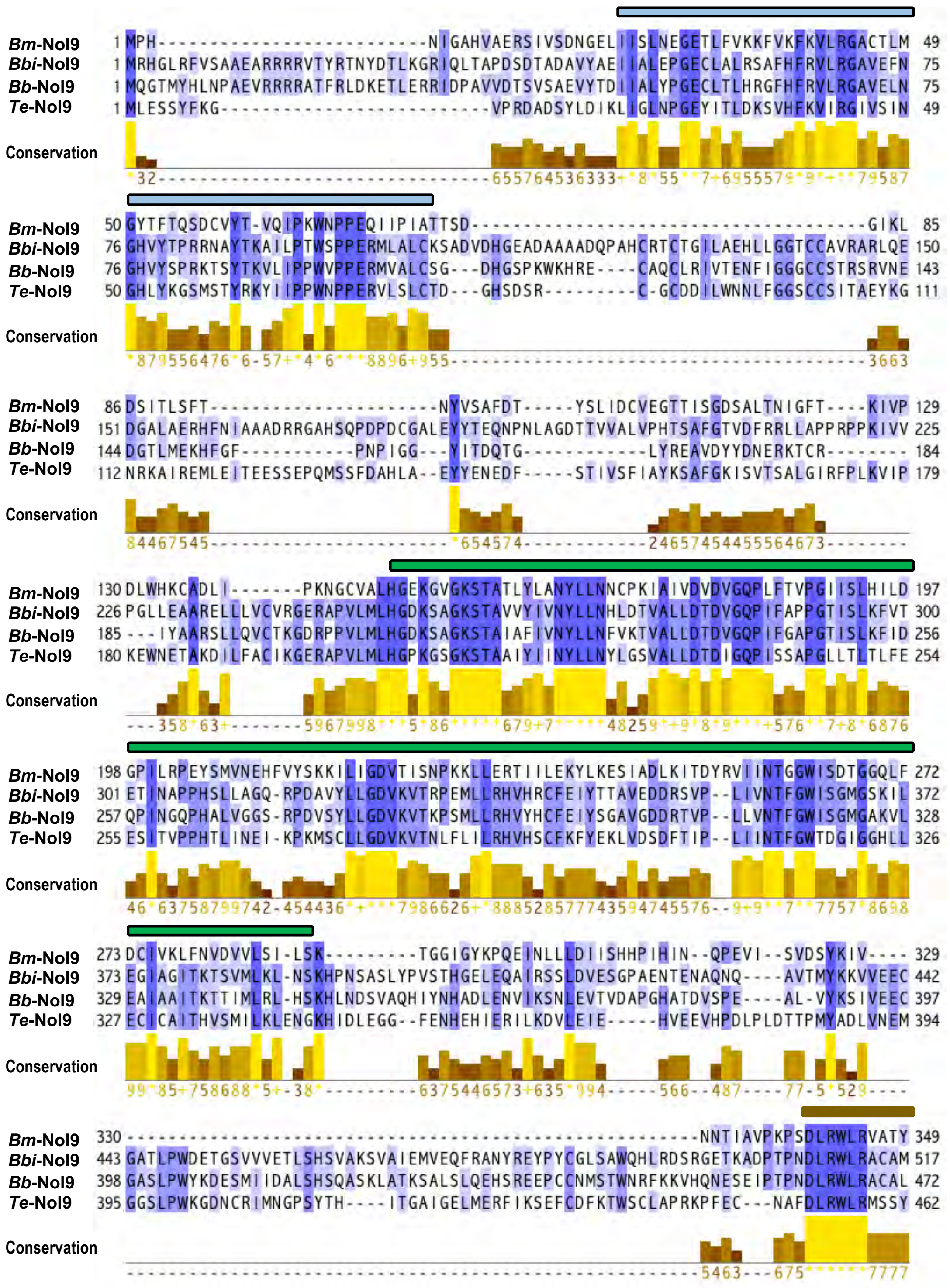

Supplementary Fig. S10

The legend for this figure is placed on the next page.

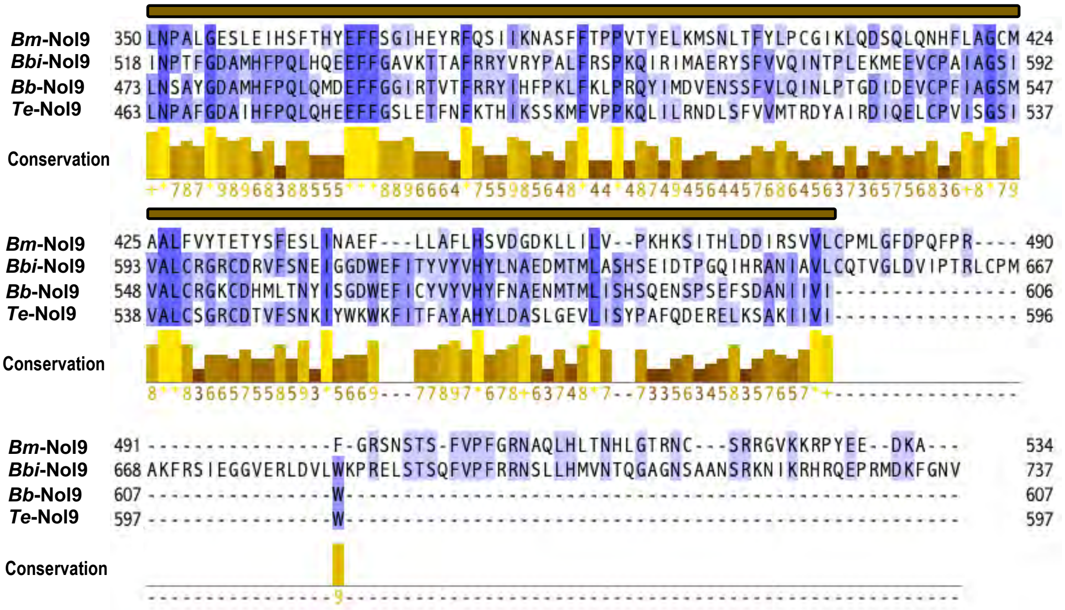

Supplementary Fig. S10 Amino acid alignment of Alveolata Nol9/Grc3 group proteins with Nol9_eN3 and Nol9_eC2 domains. Four samples of Alveolata Nol9/Grc3 group proteins (Supplementary Table S3) were used for the alignment. Rectangular boxes above the sequences indicate protein domain structures (Supplementary Table S4): Nol9_eN3 (light blue), Clp1_P (green), and Nol9_eC2 (light brown). The lower part of the figure shows the conservation scores of the alignment. See Supplementary Table S3 for species and protein information used for the analysis.

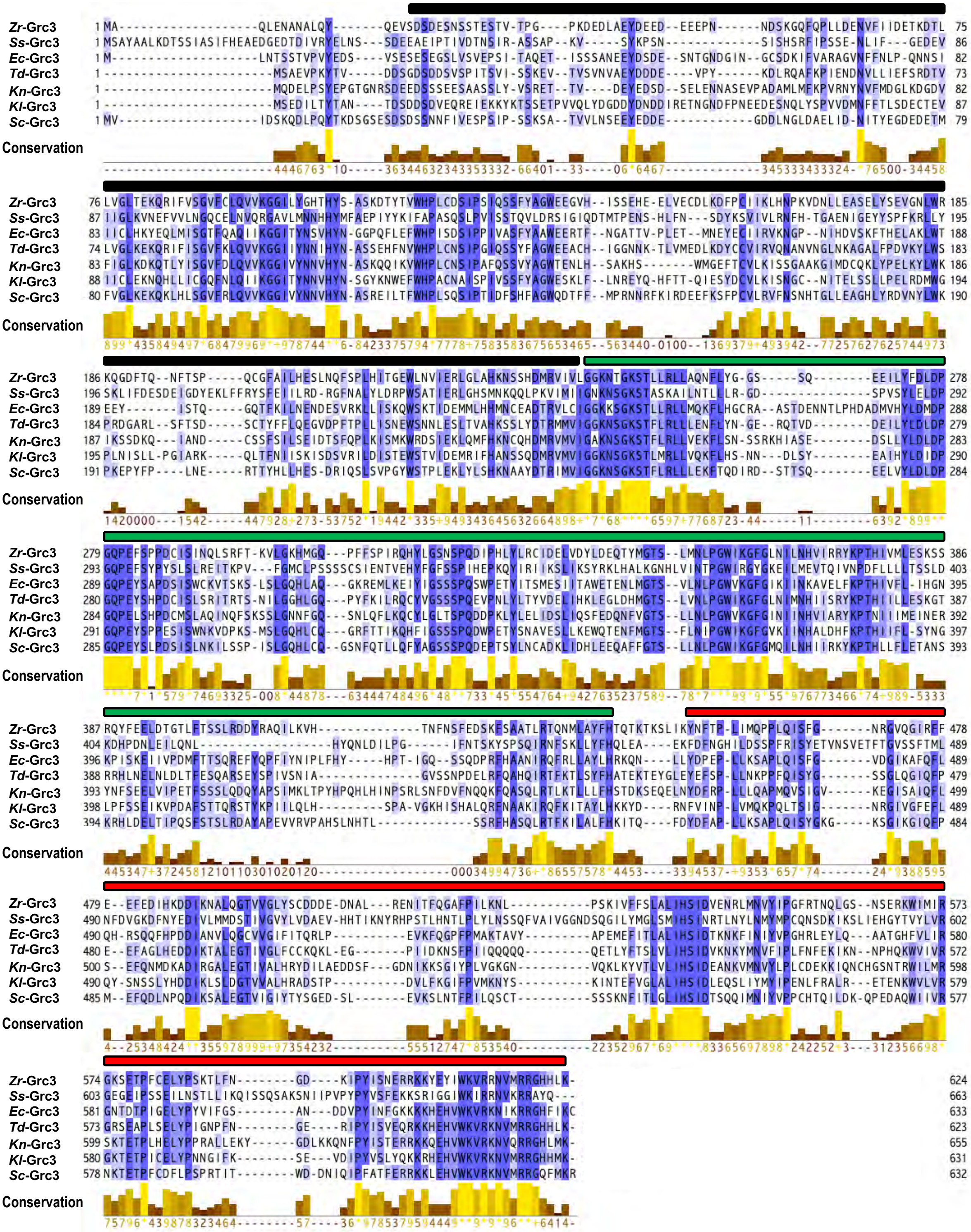

Supplementary Fig. S11 Amino acid alignment of the fungal Nol9/Grc3 group proteins with Grc3_eN1 and Grc3_eC1 domains. Seven samples of fungal Nol9/Grc3 group proteins with Grc3_eN1 and Grc3_eC1 domains (Supplementary Table S3) were used for the alignment. Rectangular boxes above the sequences indicate protein domain structures (Supplementary Table S4): Grc3_eN1 (black), Clp1_P (green), and Grc3_eC1 (red). The lower part of the figure shows the conservation scores of the alignment. See Supplementary Table S3 for species and protein information used for the analysis.

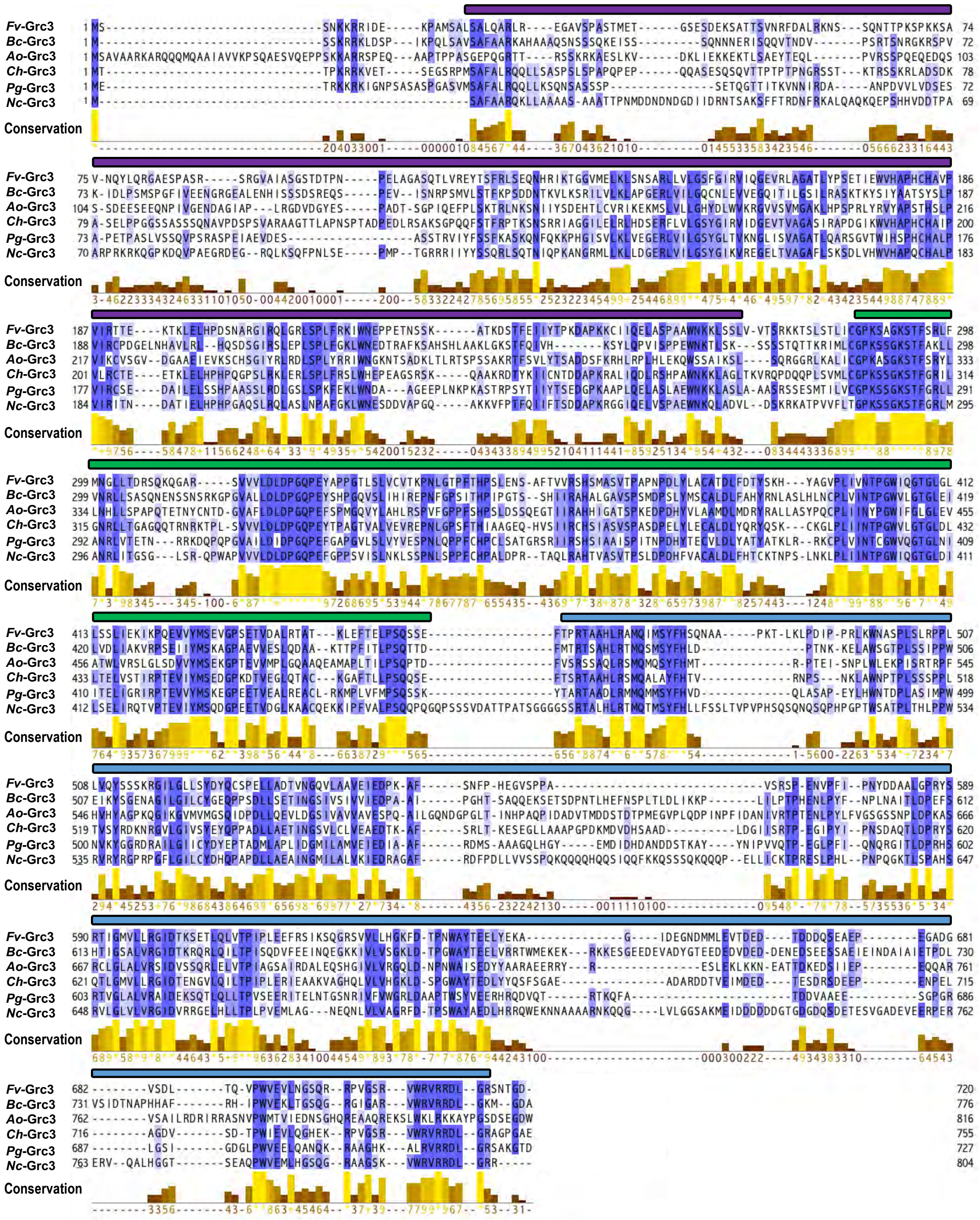

Supplementary Fig. S12 Amino acid alignment of fungal Nol9/Grc3 group proteins with Grc3_eN2 and Grc3_eC2 domains. Six examples of fungal Nol9/Grc3 group proteins containing Grc3_eN2 and Grc3_eC2 domains (Supplementary Table S3) were used for the alignment. Rectangular boxes above the sequences indicate protein domain structures (Supplementary Table S4): Grc3_eN2 (purple), Clp1_P (green), and Grc3_eC2 (blue). The lower part of the figure shows the conservation scores of the alignment. See Supplementary Table S3 for species and protein information used for the analysis.

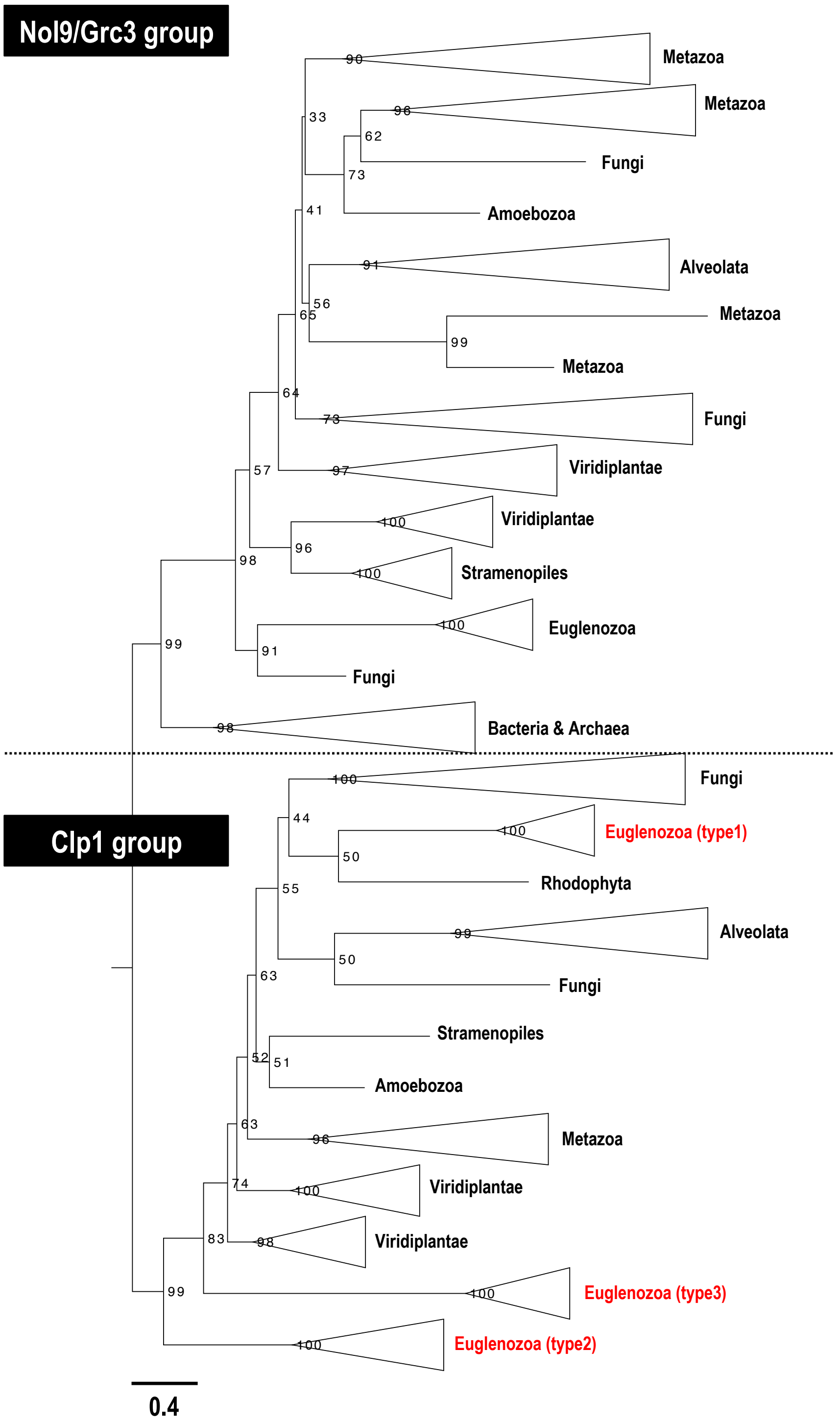

Supplementary Fig. S13 Classification of Clp1 family proteins (both Clp1 and Nol9/Grc3 groups) constructed from the amino acid sequences of the PNK domain. Clp1 family proteins were classified according to the amino acid sequence similarity of the PNK domain, which is commonly conserved among Clp1 family proteins. Among 254 Clp1 family proteins (Supplementary Table S2b), 253 Clp1 family proteins from 250 eukaryotes, two archaea, and one bacterium were used, excluding one species, *Nasonia vitripennis* (RefSeqID: XP_008207576.1), with a partially incomplete PNK domain region. The LG+R6 model was used for this phylogenetic tree. Midpoint rooting was applied during tree visualisation. The scale bar under the tree indicates the number of amino acid substitutions per site. The protein names of representative species and taxonomic groups (Kingdom to Phylum) are listed next to the phylogenetic tree. Prokaryota, Archaea, and Bacteria are described as domains. Types 1–3 are protein types of Euglenozoa Clp1 group proteins classified according to their sequence similarity. The same taxa are grouped in triangles, the sizes of which reflect the number of sequences. Numbers on the branches indicate Ultrafast bootstrap (UFBoot2) values. Horizontal dotted lines were used to divide the Clp1 and Nol9/Grc3 groups.

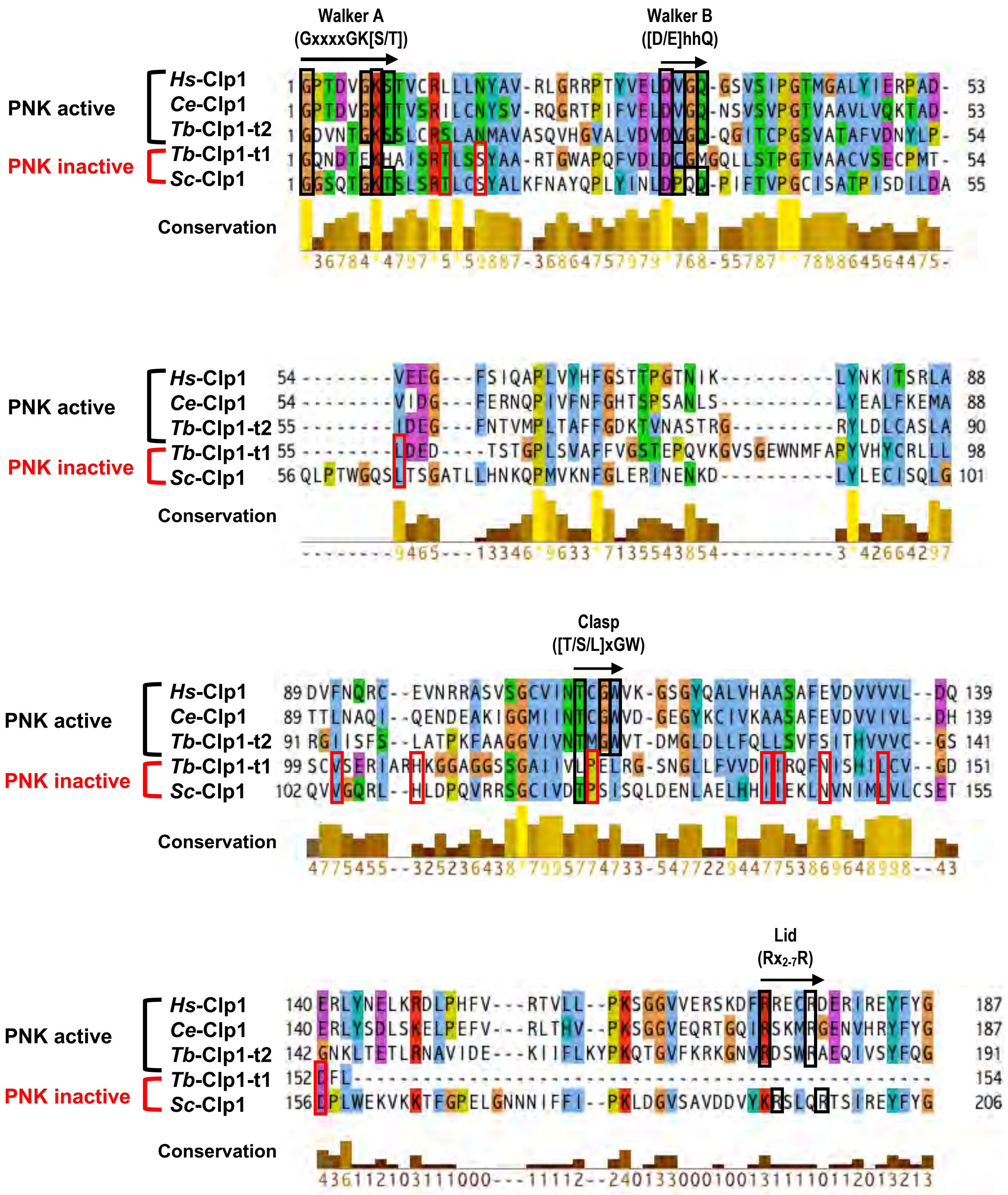

Supplementary Fig. S14 Comparison of active and inactive Clp1 amino acid sequences in the PNK domain regions of representative species. Multiple amino acid sequence alignments were performed for the PNK domain regions of 3 active Clp1 and 2 inactive Clp1 for PNK activity. The names of four motifs, Walker A, Walker B, Clasp, and Lid, and their consensus sequences are shown above the aligned sequences. Amino acid residues conserved within the motif are enclosed in black boxes, and amino acid residues commonly conserved only in inactive Clp1 are enclosed in red boxes. The color of each amino acid residue was the color specified in Clustal X according to its chemical nature. Abbreviations are as follows: *Hs*-Clp1, *Homo sapiens*-Clp1; *Ce*-Clp1, *Caenorhabditis elegans*-Clp1; *Tb*-Clp1-t2, *Trypanosoma burucei*-Clp1 type2; *Tb*-Clp1-t1, *Trypanosoma burucei*-Clp1 type1; *Sc*-Clp1, *Saccharomyces cerevisiae*-Clp1.

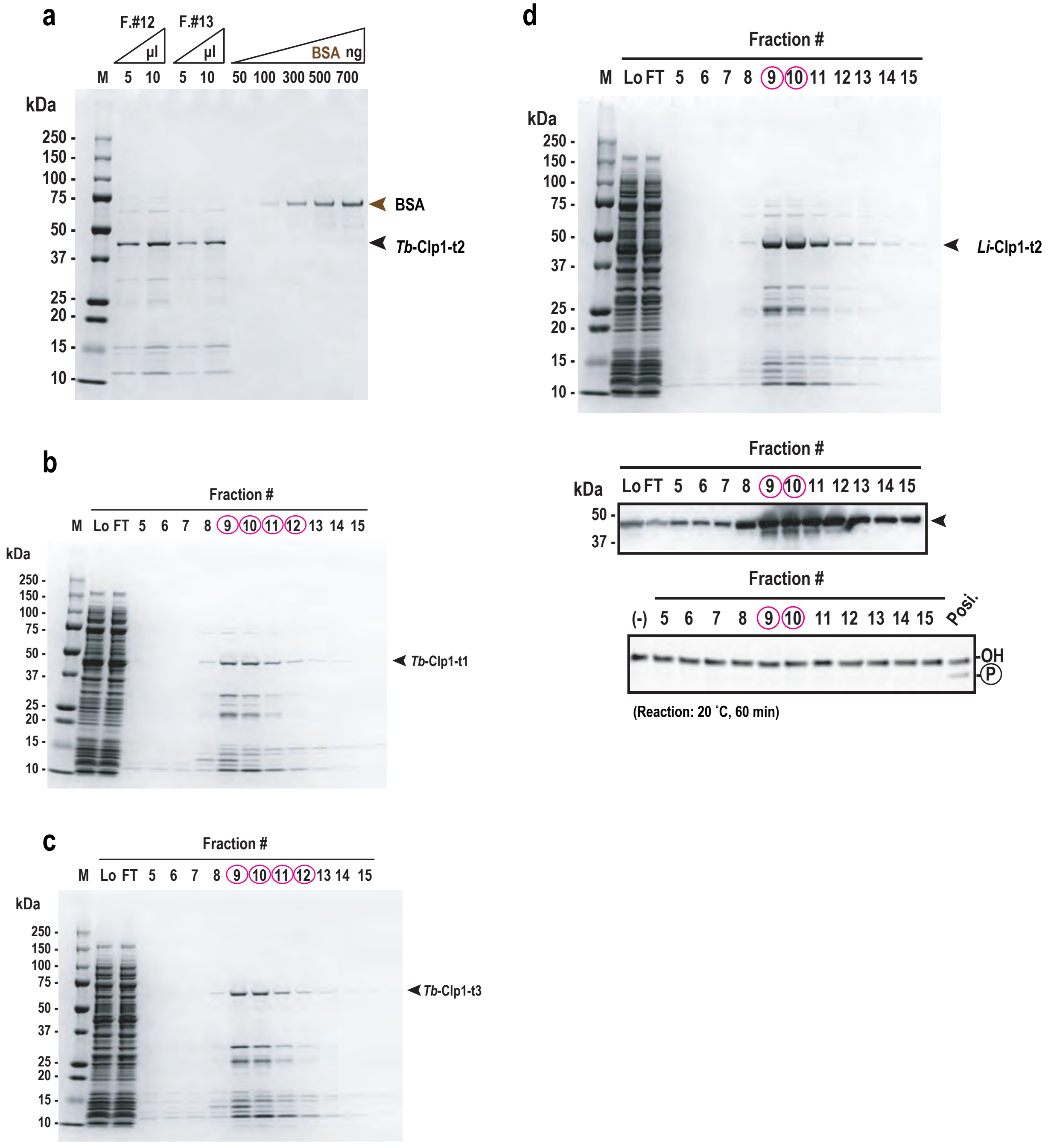

Supplementary Fig. S15 Purification of recombinant Euglenozoa Clp1 group proteins and their PNK activities (supporting data). (a) Determination of concentration of partially purified *Tb*-Clp1-t2 protein using bovine serum albumin (BSA) at known concentrations (50–700 ng) as the standard. Samples were subjected to 10%–20% SDS-PAGE and stained with Coomassie Brilliant Blue. F.: fraction. (b) Each fraction of the recombinant *Tb*-Clp1-t1 and (c) *Tb*-Clp1-t3 proteins purified with HisTrap HP affinity chromatography was subjected to 10%–20% SDS-PAGE and stained with Coomassie Brilliant Blue. (d) Each fraction of recombinant *Li*-Clp1-t2 protein purified with HisTrap HP affinity chromatography was subjected to 10%–20% SDS-PAGE and stained with Coomassie Brilliant Blue (top). Western blotting analysis with anti-His-tag antibody (middle), and PNK activity using ssRNA as the substrate (bottom). Recombinant *Tb*-Clp1-t2 protein was used as the positive control. Protein peaks on column chromatography are indicated by red circles. Arrows indicate the position of each protein (BSA, light brown; each recombinant protein. black).

References

Chan PP, Lowe TM. 2009. GtRNAdb: a database of transfer RNA genes detected in genomic sequence. Nucleic Acids Res 37: D93-97.

Saito M, Sato A, Nagata S, Tamaki S, Tomita M, Suzuki H, Kanai A. 2019. Large-Scale Molecular Evolutionary Analysis Uncovers a Variety of Polynucleotide Kinase Clp1 Family Proteins in the Three Domains of Life. Genome Biol Evol 11: 2713-2726.
